# Extensive antibiotic prescription rate among hospitalized patients in Uganda: but with frequent missed-dose days

**DOI:** 10.1093/jac/dkw025

**Published:** 2016-03-05

**Authors:** Ronald Kiguba, Charles Karamagi, Sheila M. Bird

**Affiliations:** 1 Department of Pharmacology and Therapeutics, Makerere University College of Health Sciences, Kampala, Uganda; 2 Clinical Epidemiology Unit, Makerere University College of Health Sciences, Kampala, Uganda; 3 Medical Research Council Biostatistics Unit, Cambridge, UK

## Abstract

**Objectives:**

To describe the patterns of systemic antibiotic use and missed-dose days and detail the prescription, dispensing and administration of frequently used hospital-initiated antibiotics among Ugandan inpatients.

**Methods:**

This was a prospective cohort of consented adult inpatients admitted on the medical and gynaecological wards of the 1790 bed Mulago National Referral Hospital.

**Results:**

Overall, 79% (603/762; 95% CI: 76%–82%) of inpatients received at least one antibiotic during hospitalization while 39% (300/762; 95% CI: 36%–43%) had used at least one antibiotic in the 4 weeks pre-admission; 1985 antibiotic DDDs, half administered parenterally, were consumed in 3741 inpatient-days. Two-fifths of inpatients who received at least one of the five frequently used hospital-initiated antibiotics (ceftriaxone, metronidazole, ciprofloxacin, amoxicillin and azithromycin) missed at least one antibiotic dose-day (44%, 243/558). The per-day risk of missed antibiotic administration was greatest on day 1: ceftriaxone (36%, 143/398), metronidazole (27%, 67/245), ciprofloxacin (34%, 39/114) and all inpatients who missed at least one dose-day of prescribed amoxicillin and azithromycin. Most patients received fewer doses than were prescribed: ceftriaxone (74%, 273/371), ciprofloxacin (90%, 94/105) and metronidazole (97%, 222/230). Of prescribed doses, only 62% of ceftriaxone doses (1178/1895), 35% of ciprofloxacin doses (396/1130) and 27% of metronidazole doses (1043/3862) were administered. Seven percent (13/188) of patients on intravenous metronidazole and 6% (5/87) on intravenous ciprofloxacin switched to oral route.

**Conclusions:**

High rates of antibiotic use both pre-admission and during hospitalization were observed, with low parenteral/oral switch of hospital-initiated antibiotics. Underadministration of prescribed antibiotics was common, especially on the day of prescription, risking loss of efficacy and antibiotic resistance.

## Introduction

Worldwide use of antibiotics, pharmacologically classified as antibacterial agents,^1^ increased by 36% in healthcare in the first decade of the new millennium.^2^ Indiscriminate use may have contributed to this upward trend.^3,4^ Inappropriate prescribing, dispensing and administration of systemic antibiotics undermines their utility and cost-effectiveness and increases the risk of suspected adverse drug reactions (ADRs),^5,6^ including serious suspected ADRs,^7,8^ and antibiotic resistance.^5^

Resistance to single antibiotic agents may render entire antibiotic classes ineffective.^5^ Antibiotic resistance contributes to increased morbidity and mortality: the EU (population: 500 million) estimates up to 25 000 deaths annually^5^ and the USA (population: 319 million)^9^ estimates up to 23 000 deaths annually.^10^ Similar data are lacking in the developing world.

For almost three decades, hardly any new antibiotic classes have been discovered to combat resistance.^5,11,12^ Thus, healthcare professionals (HCPs) must preserve the effectiveness of currently available antibiotics through rational prescribing, dispensing, administration and monitoring of these medicines^5^ and by promoting their proper use by patients.

In sub-Saharan Africa, there is a paucity of published literature on the prescribing, dispensing and administration of systemic antibiotics to inpatients. Recent global estimates for antibiotic consumption did not include data from the East African region.^2^ Yet, if made available, such data could enhance future strategies for improving antibiotic use and combating resistance in resource-limited settings.^13^

In Uganda, decisions by HCPs to prescribe systemic antibiotics to inpatients are often based on unconfirmed diagnosis. Evidence-based prescribing and dispensing of antibiotics should be the standard,^14^ but the lack of rapid diagnostic tools is a limitation.^13^ Little is known about the patterns of systemic antibiotic use by hospitalized Ugandan patients. It is not known e.g. whether hospitalized patients receive correct prescriptions of systemic antibiotics or whether patients complete full courses of prescribed antibiotics (e.g. if discharged prior to receipt of all prescribed parenteral doses).

We therefore describe the pattern of systemic antibiotic use by DDDs, antibiotic class, individual antibiotic, missed-dose days and parenteral/oral switch. We also provide an account of the prescription, dispensing and administration of frequently used hospital-initiated systemic antibiotics (ceftriaxone, metronidazole, ciprofloxacin, amoxicillin and azithromycin) among hospitalized Ugandan patients admitted on the medical and gynaecological wards of Mulago National Referral Hospital.

## Methods

### Study design and setting

We conducted a prospective cohort study among hospitalized patients, ≥18 years of age, at the 1790 bed Mulago National Referral Hospital^15^ where the annual turnover exceeds 140 000 inpatients. The study setting comprised three medical wards [Infectious Diseases and Gastrointestinal Illnesses (IDGI), Haematology, Neurology and Endocrinology (HNE) and Cardiovascular, Pulmonology and Nephrology (CPN)] and one Gynaecology (GYN) ward. Each of the four wards has an official bed capacity of 54, but can receive 70–80 admissions. Admissions on the medical wards average 10–15 patients per day in each of wards IDGI and CPN and 5–10 patients per day in the HNE ward, thus about 25–40 medical wards admissions per day; and 20–25 admissions per day on the GYN ward.

The process of medication ordering and administration is a hand-written system whereby doctors prescribe medicines and transcribe medication orders onto patients' treatment/administration charts. Prescribed injectable antibiotics are dispensed to patients by ward pharmacists/pharmacy technicians in amounts that are sufficient for 1 or 2 days of treatment. Patients/caregivers are expected to refill prescriptions at ward pharmacies sufficiently early to avoid missed medication doses. Controlled dispensing, as described, is to avoid misuse of on-ward prescribed medicines. Key/essential medicines (e.g. injectable ceftriaxone) are stocked in small amounts by ward nurses for emergencies. If in stock, prescribed medicines are provided free of charge to patients; otherwise, patients have to purchase them from private community pharmacies. Nurses urge patients to take their prescribed oral or topical medication, but directly administer parenteral medicines and record this information (drug name, dose, route and time of administration) on patients' hospital medication administration charts.

### Data collection

During October to November 2013, a pilot phase was conducted on all four wards to assess the feasibility of undertaking the cohort study and to refine study instruments. The pilot data, however, are excluded from the final results presented in this paper. The main study commenced in December 2013 to April 2014, when research teams recruited and followed up patients on the study wards according to a systematic random sampling procedure whereby three new admissions per day on long-stay wards (HNE/CPN) and six per day on short-stay wards (IDGI/GYN) were to be recruited. Each ward team purposed to select at random one of the first two (IDGI), three (HNE) and four (CPN/GYN) new admissions and thereafter every second, third and fourth admission, respectively.

In practice, however, it was difficult to implement systematic random sampling because of the following reasons: (i) the sampling frames (ward registers) were not always reliable since registration of new patients into the study wards was not always done immediately on admission (hence, the registers were sometimes not up to date when the research teams needed to use them to identify and approach new study patients); (ii) selected registered patients were sometimes unavailable on their hospital beds at the time of recruitment; (iii) patients were too ill to cooperate; (iv) patients declined to give informed consent when research teams approached them; (v) ward admissions were irregular, ranging from zero to rather high admission rates that would outpace systematic random sampling by the research teams; and (vi) routine minor and major ward rounds interrupted recruitment of new study patients since patient engagement was not permitted during ward rounds, which were sometimes rather lengthy. Given these limitations, we modified the sampling approach from just using the registers to also actively looking for newly admitted patients who, although admitted, were not yet recorded in the ward register(s). Delays in patient registration usually occurred immediately after weekends (on Mondays and Tuesdays) when patient admission rates frequently exceeded the capacity/work rate of the ward staff. However, the patients were typically registered within 24 h after admission.

Voluntary participation of patients was sought through provision of written informed consent. Consenting and recruiting a new patient took between 1 and 2 h, sometimes longer, while the daily mean time burden of patient contact with a research team was estimated at 10–30 min.

Research assistants were trained intensively for a 1 week period on the practical pharmacovigilance aspects of the project and thereafter R. K. conducted daily reviews of study procedures to ensure adherence to the study protocol (see the Supplementary Methods, available as Supplementary data at *JAC* Online).

Four research teams collected the data. Each team comprised a medical doctor (clinician), pharmacist and degree nurse. Ward-based physicians, all staff of Mulago National Referral Hospital (one physician based on the medical wards and another on the GYN ward), served as study physicians to resolve any clinical problems encountered by the data collection teams, while R. K. resolved pharmacological issues.

On recruitment (day 1), each research team conducted baseline assessment of the consented patients to obtain relevant data on demographics, clinical conditions and medications and thereafter conducted daily assessments until discharge, transfer, death or loss to follow-up. A 26 page case report form (CRF) was used to capture both baseline and daily follow-up patient information (see the Supplementary Methods for further details on data collection). For example, medication data were obtained from the patient's hospital file (clinical notes, treatment sheets and drug administration charts), dispensing records of ward pharmacies, pill count validation of a patient's oral medication (tablets, capsules) and by viewing of unused injectable medicine vials/ampoules in the possession of the patient/caregiver.

Research teams collected data daily from 8.00am to 6.00pm from Monday to Friday and from 10.00am to 6.00pm on weekends and public holidays.

### Data management

Given the large amount of data collected, R. K. and S. M. B. adopted an efficient data entry design that accorded with planned statistical analyses. Key variables for initial capture were identified (demographics, relevant baseline data on medication history and clinical condition). Data for the 762 cases were manually abstracted by R. K. from the CRFs onto data abstraction forms. See the Supplementary Methods for further details on data management.

### Antibiotic classification

Using the WHO Anatomical Therapeutic Chemical (ATC) classification system,^16^ antibiotics are defined as antibacterial agents for systemic use (J01) and include dapsone (J04BA02) and oral nitroimidazole derivatives (P01AB). Dapsone was prescribed mainly for prophylaxis against opportunistic infections^17^ in HIV-positive patients who could not tolerate co-trimoxazole. Topical (ophthalmic, otic, dermatologic or vaginal) antibiotics and other antimycobacterial agents (ATC group J04) were excluded.

### Statistical analysis

#### Patterns of antibiotic use

The proportion of patients who received any antibiotic prior to admission and/or during their hospital stay was computed before and after excluding: (i) co-trimoxazole use alone; or (ii) co-trimoxazole and dapsone use (both used for prophylaxis against HIV/AIDS-related opportunistic infections). Similarly, we computed proportions of patients who were switched from the intravenous to the oral route of antibiotic administration and those who experienced prescription errors and missed antibiotic dose-days. We used the ATC/DDD index^16^ to convert administered doses (in grams or mega units) of each antibiotic and route of administration into DDDs (see Table S1). We standardized antibiotic use into DDDs per 1000 patient-days for each antibiotic and computed overall antibiotic use in DDDs per 1000 patient-days. Patient-days were calculated by summing the number of days of hospital stay contributed by each studied inpatient. For example, if a patient was admitted on 1 March 2014 and discharged on 3 March 2014, the patient would contribute three patient-days. We also computed antibiotic DDDs per 100 hospital admissions.

To permit analysis of antibiotic use by clinical condition, working diagnoses were classified into 10 diagnostic groups using the *International Classification of Diseases, Tenth Revision, Clinical Modification (ICD-10-CM)* diagnosis codes as a guide,^18^ namely: malaria, immunosuppressed syndrome (ISS) or HIV/AIDS, TB, respiratory tract conditions excluding TB, skin conditions, gastrointestinal tract conditions, genitourinary tract conditions, chronic/comorbid conditions, miscellaneous infections and all other diagnoses. Respiratory, gastrointestinal and genitourinary tract conditions were subcategorized into: (i) infection-related diagnoses; and (ii) other conditions. One or more working diagnosis(es)/diagnosis group(s) could occur in any individual inpatient.

χ^2^ tests were used to assess univariate-level relationships between patient characteristics and antibiotic use (yes/no); also ORs with their 95% CIs. Poisson CIs were used for counts <16. Stata 12.0^19^ was used for all statistical analyses.

#### Identification of missed antibiotic dose-days

Some patients were prescribed an antibiotic that they did not receive. We account for them separately. For patients who were prescribed each of the five frequently administered hospital-initiated individual antibiotics (ceftriaxone, metronidazole, ciprofloxacin, amoxicillin and azithromycin) and received at least one dose, we determined the number of missed-dose days of the individual antibiotic(s) per patient, as detailed in Figure S1. Detailed analyses of co-trimoxazole prescription, dispensing and administration were excluded since most patients had commenced co-trimoxazole use several weeks, months or years pre-admission.

### Ethics clearance

We obtained ethics approval for the study from the School of Medicine Research and Ethics Committee, Makerere University College of Health Sciences (REC REF no. 2011-113), the Mulago Hospital Research and Ethics Committee (MREC 253) and the Uganda National Council for Science and Technology (HS 1151).

## Results

### Study population

We studied 762 patients, 70% of whom were female (534/762; 95% CI: 67%–73%): all GYN ward patients (*n* = 191) and 60% otherwise (343/571; 95% CI: 56%–64%). Patients' mean age was 34.8 years (SD = 14.8). Patients spent 3741 patient-days in hospital with median length of hospital stay of 4 (IQR: 3–6) days. Thirty percent of patients were known to be HIV positive (232/762) and 30% had been hospitalized in the previous 3 months (230/762) (see Table 1), including 32% (75/232) of known HIV-positive patients.

**Table 1.** Demographic and clinical characteristics of 762 hospitalized patients, Uganda, 2014

**Characteristics**						
Age (years), mean (SD)/median (IQR)			34.8 (SD = 14.8, *n* = 762)/30 (24–42)			
Length of hospital stay (days), mean (SD)/median (IQR)			4.9 (SD = 2.9, *n* = 762)/4 (3–6)			
Patient-days of observation, overall			3741			
**Extent of antibiotic use**						
	Antibiotic use, *n* (%)					
	yes	no	total			
Antibiotic use pre-admission	300 (39)	462 (61)	762			
Antibiotic use during hospitalization^a^	603 (79)	159 (21)	762			
no co-trimoxazole use pre-admission	472 (75)	159 (25)	631			
no in-hospital co-trimoxazole and dapsone use	436 (73)	159 (27)	595			
Antibiotic use either pre-admission or during hospitalization	629 (83)	133 (17)	762			
**Subgroup analyses on key variables**						
	Antibiotic use, *n* (%)			Single factor analysis		
	yes	no	total (% column)	OR	95% CI for OR	*P*
Gender						
male	190 (83)	38 (17)	228 (30)	1.0		
female	413 (77)	121 (23)	534 (70)	0.7	0.46–1.02	0.063
Ward						
GYN	129 (68)	62 (32)	191 (25)	1.0		
IDGI	273 (85)	47 (15)	320 (42)	2.8	1.81–4.30	<0.001
HNE	88 (75)	29 (25)	117 (15)	1.5	0.87–2.45	0.153
CPN	113 (84)	21 (16)	134 (18)	2.6	1.48–4.51	0.001
Number of working diagnoses						
1	84 (61)	54 (39)	138 (18)	1.0		
2	162 (78)	46 (22)	208 (27)	2.3	1.41–3.63	0.001
3	155 (83)	31 (17)	186 (24)	3.2	1.92–5.38	<0.001
≥4	202 (88)	28 (12)	230 (30)	4.6	2.75–7.82	<0.001
Changes to working diagnoses versus discharge diagnoses						
0	160 (68)	77 (32)	237 (31)	1.0		
1	291 (84)	57 (16)	348 (46)	2.5	1.66–3.64	<0.001
≥2	152 (86)	25 (14)	177 (23)	2.9	1.77–4.84	<0.001
Length of hospital stay						
<5 days	308 (71)	124 (29)	432 (57)	1.0		
≥5 days	295 (89)	35 (11)	330 (43)	3.4	2.26–5.10	<0.001
HIV serostatus						
negative	242 (71)	98 (29)	340 (45)	1.0		
positive	221 (95)	11 (5)	232 (30)	8.1	4.25–15.6	<0.001
unknown	140 (74)	50 (26)	190 (25)	1.1	0.76–1.69	0.537
Hospitalization in previous 3 months						
no	419 (79)	113 (21)	532 (70)	1.0		
yes	184 (80)	46 (20)	230 (30)	1.1	0.73–1.58	0.699
Major working diagnoses						
respiratory tract conditions (113/130 include infections)						
no	479 (76)	153 (24)	632 (83)	1.0		
yes	124 (95)	6 (5)	130 (17)	6.6	2.85–15.3	<0.001
gastrointestinal tract conditions (150/193 include infections)						
no	431 (76)	138 (24)	569 (75)	1.0		
yes	172 (89)	21 (11)	193 (25)	2.6	1.60–4.29	<0.001
genitourinary tract conditions (81/104 include infections)						
no	518 (79)	140 (21)	658 (86)	1.0		
yes	85 (82)	19 (18)	104 (14)	1.2	0.71–2.06	0.484
skin conditions						
no	587 (79)	159 (21)	746 (98)	1.0		
yes	16 (100)	0 (0)	16 (2)	infinite		
malaria						
no	502 (81)	119 (19)	621 (81)	1.0		
yes	101 (72)	40 (28)	141 (19)	0.6	0.39–0.91	0.016
ISS or HIV/AIDS^b^						
no	456 (75)	154 (25)	610 (80)	1.0		
yes	147 (97)	5 (3)	152 (20)	9.9	3.99–24.7	<0.001
TB						
no	485 (76)	155 (24)	640 (84)	1.0		
yes	118 (97)	4 (3)	122 (16)	9.4	3.42–26.0	<0.001
chronic/comorbid conditions						
no	302 (79)	82 (21)	384 (50)	1.0		
yes	301 (80)	77 (20)	378 (50)	1.1	0.75–1.51	0.738
miscellaneous infections						
no	498 (77)	150 (23)	648 (85)	1.0		
yes	105 (92)	9 (8)	114 (15)	3.5	1.74–7.11	<0.001
other conditions						
no	533 (79)	143 (21)	676 (89)	1.0		
yes	70 (81)	16 (19)	86 (11)	1.2	0.66–2.08	0.584

^a^Eighty-one percent (131/162) of patients who received co-trimoxazole during hospital stay had received it during the month preceding hospitalization. Overall, 167 patients used either co-trimoxazole (162) or dapsone (5) for prophylaxis against opportunistic infections.

^b^Not all HIV-positive patients had ISS.

### Extent of antibiotic use

Seventy-nine percent (603/762; 95% CI: 76%–82%) of hospitalized patients received at least one antibiotic during their hospital stay while 39% (300/762; 95% CI: 36%–43%) used at least one antibiotic in the 4 weeks pre-admission. Twenty-one percent of patients (162/762; 95% CI: 18%–24%) used co-trimoxazole during hospital stay, 159 of them known to be HIV positive, of whom 81% (131/162) had received co-trimoxazole during the month preceding hospitalization. Excluding this group, 75% (472/631; 95% CI: 71%–78%) of patients who reported that they did not use co-trimoxazole during the month preceding hospitalization received at least one antibiotic initiated during their hospital stay. The proportion of patients who used at least one antibiotic during hospitalization increased with the number of working diagnoses: single diagnosis (61%, 84/138; 95% CI: 52%–69%), two diagnoses (78%, 162/208; 95% CI: 72%–84%), three diagnoses (83%, 155/186; 95% CI: 78%–89%) and four or more diagnoses (88%, 202/230; 95% CI: 83%–92%). Hospital-initiated antibiotic use was 68% (160/237; 95% CI: 62%–74%) for patients with no changes to working diagnoses from admission to discharge and 84% otherwise (443/525; 95% CI: 81%–87%). See Table 1 also for other major working diagnoses for which antibiotics were administered.

### Antibiotic use by DDDs and antibiotic class

Overall, 1985 systemic antibiotic DDDs, half administered parenterally (48%, 960/1985), were consumed during 3741 in-hospital patient-days, i.e. 531 DDDs per 1000 patient-days or 261 DDDs per 100 hospital admissions. Commonly used antibiotic classes by percentage DDDs were: cephalosporins (21%), combinations of sulphonamides with trimethoprim (18%), fluoroquinolones (17%), imidazole derivatives (16%), macrolides, lincosamides and streptogramins (13%) and penicillins (11%) (see Table 2 and Table S2).

**Table 2.** Patterns of systemic antibiotic use among 762 hospitalized patients, Uganda, 2014

	Oral	Intravenous/ intramuscular	No. of patients	Patterns of systemic antibiotic use			
				DDDs	% DDDs	DDDs/1000 patient-days	DDDs/100 admissions
Antibiotic name							
cephalosporins J01D	5	398	403	409.5	21	109.5	53.7
combinations of sulphonamides with trimethoprim J01EE	162	0	162	358.0	18	95.7	47.0
fluoroquinolones J01MA	33	99	132	342.9	17	91.7	45.0
imidazole derivatives J01XD^a^	60	188	248	311.4	16	83.3	40.9
macrolides, lincosamides and streptogramins J01F	56	1	57	248.5	13	66.5	32.6
penicillins J01C	84	28	112	227.4	11	60.8	29.8
antimycobacterials J04B	5	0	5	56.0	3	15.0	7.3
aminoglycosides J01G	0	11	11	15.0	1	4.0	2.0
tetracyclines J01A	5	0	5	16.0	1	4.3	2.1
carbapenems J01DH	0	1	1	0.3	0	0.1	0.0
total				1985.0		530.9	260.5
Route of antibiotic administration							
oral			112	1024.8	52	274.1	134.5
parenteral (intravenous/intramuscular)			491	960.2	48	256.8	126.0
total			603	1985.0	100	530.9	260.5

^a^Parenteral formulations of nitroimidazoles are classified as J01XD and oral formulations as P01AB in the WHO ATC/DDD index.

### Frequently used individual antibiotics

The most frequently used individual antibiotics, by percentage of patients using antibiotics, were ceftriaxone (66%, 398/603; 95% CI: 62%–70%), metronidazole (41%, 246/603; 95% CI: 37%–45%), co-trimoxazole (27%, 162/603; 95% CI: 23%–31%), ciprofloxacin (19%, 114/603; 95% CI: 16%–22%) and amoxicillin (10%, 57/603; 95% CI: 7%–12%) (see Table 3). When standardized by percentage DDDs, the most commonly used individual antibiotics were ceftriaxone (20%), co-trimoxazole (18%), metronidazole (16%) and ciprofloxacin (14%), followed by amoxicillin (9%) and azithromycin (6%) (see Table S2).

**Table 3.** Frequency of antibiotic use among 603 out of 762 patients who used antibiotics during hospitalization, Uganda, 2014

Individual antibiotic	Oral	Intravenous	Number (%) of patients who received the antibiotic
Ceftriaxone	0	398	398 (66.0)
Metronidazole^a^	58	188	246 (40.8)
Co-trimoxazole	162	0	162 (26.9)
Ciprofloxacin	27	87	114 (18.9)
Amoxicillin	57	0	57 (9.5)
Ampicillin/cloxacillin	19	13	32 (5.3)
Azithromycin	26	0	26 (4.3)
Erythromycin	19	0	19 (3.2)
Levofloxacin	5	12	17 (2.8)
Gentamicin	0	11	11 (1.8)
Clarithromycin	11	0	11 (1.8)
Ampicillin	0	11	11 (1.8)
Amoxicillin/clavulanate	8	0	8 (1.3)
Other	18	6	24 (4.0)

^a^Overall, 246 patients received metronidazole but one patient did not have details.

### Missed-dose days of five frequently used hospital-initiated antibiotics

#### Ceftriaxone

Forty-three percent (171/398; 95% CI: 38%–48%) of patients who received intravenous ceftriaxone missed at least one dose-day of ceftriaxone. The per-day risk of missed ceftriaxone administration was greatest on day 1 of prescribed treatment (36%, 143/398; 95% CI: 31%–41%) (see Table S3). Moreover, 26% (105/398; 95% CI: 22%–31%) received only one dose-day of ceftriaxone treatment; or 21% (42/200; 95% CI: 16%–27%) of the patients prescribed a 5 day course of ceftriaxone.

#### Metronidazole

Thirty-one percent (77/245; 95% CI: 25%–37%) of patients who received oral or intravenous metronidazole missed at least one dose-day of metronidazole. The per-day risk of missed metronidazole administration was greatest on day 1 of prescribed treatment (27%, 67/245; 95% CI: 21%–33%) (see Table S3); 26% (63/245; 95% CI: 20%–32%) received only one dose-day of metronidazole treatment; or 24% (31/131; 95% CI: 17%–32%) of the patients prescribed a 5 day course of metronidazole.

#### Ciprofloxacin

Thirty-eight percent (43/114; 95% CI: 29%–47%) of patients who received oral or intravenous ciprofloxacin missed at least one dose-day. The per-day risk of missed ciprofloxacin administration was greatest on day 1 (34%, 39/114; 95% CI: 25%–43%) (see Table S3); 29% (33/114; 95% CI: 21%–38%) received only one dose-day of ciprofloxacin treatment; or 26% (25/98; 95% CI: 17%–35%) of the patients prescribed a 5 day course of ciprofloxacin.

#### Amoxicillin

Eleven percent (6/57; 95% CI: 3%–18%) of patients who received oral amoxicillin missed one dose-day of treatment, all of whom missed day 1 (see Table S3). Most patients (93%, 53/57) received the amoxicillin towards discharge from the ward and 26% (15/57; 95% CI: 15%–43%) received only one dose-day of amoxicillin.

#### Azithromycin

Thirty-one percent (8/26; 95% CI: 13%–61%) of patients who received oral azithromycin missed at least one dose-day of treatment, all of whom missed day 1 (see Table S3), and 27% (7/26; 95% CI: 11%–55%) received only one dose-day of azithromycin.

### Summary of missed antibiotic dose-days

Overall, 73% (558/762; 95% CI: 70%–76%) of patients in the cohort used at least one of the five frequently administered hospital-initiated antibiotics (ceftriaxone, metronidazole, ciprofloxacin, amoxicillin and azithromycin), 44% (243/558; 95% CI: 39%–48%) of whom missed at least one dose-day of antibiotic treatment.

### Administration of frequently prescribed hospital-initiated antibiotics

Of prescribed doses, only 62% of ceftriaxone doses (1178/1895), 35% of ciprofloxacin doses (396/1130) and 27% of metronidazole doses (1043/3862) were administered (see Table 4). Most patients received fewer doses than were prescribed: ceftriaxone (74%, 273/371), ciprofloxacin (90%, 94/105) and metronidazole (97%, 222/230) (see Table 5). Medication errors were observed in half (13/26) of the patients who received oral azithromycin. Seven of the 26 (27%) patients purchased the azithromycin from a private community pharmacy. Six medication administration errors were committed by patients, one medication dispensing error by a ward pharmacist and one prescription error by a medical doctor. See Tables S3 and S4 and the Supplementary Results for details on prescription, dispensing and administration.

**Table 4.** Prescribed, dispensed and administered doses of the three most frequently used hospital-initiated antibiotics among inpatients, Uganda, 2014

Individual antibiotic	Number of patients	Patient-days (mean)	Overall number of antibiotic doses (median, IQR)		
			prescribed	dispensed	administered
Ceftriaxone^a^	398	2174 (5.5)	1895 (5, 5–5)	783 (2, 1–3)	1178 (3, 1–3)
Metronidazole^a,b^	245	1240 (5.1)	3862 (15, 9–18)	1642 (4, 2–12)	1043 (3, 2–6)
Ciprofloxacin^a^	114	666 (5.8)	1130 (10, 10–11)	728 (6, 3–10)	396 (3, 1–4)

^a^Administered doses as a percentage of prescribed doses: ceftriaxone (62%, 1178/1895), metronidazole (27%, 1043/3862) and ciprofloxacin (35%, 396/1130).

^b^Overall, 246 patients received metronidazole, but one patient did not have details.

**Table 5.** Proportion of inpatients who received the full course, fewer doses or more doses of the three most frequently used hospital-initiated antibiotics, Uganda, 2014

Individual antibiotic	Number of patients^a^	Number of patients receiving antibiotics (%)		
		full course	fewer doses	more doses
Ceftriaxone	371	60 (16)	273 (74)	38 (10)
Metronidazole	230	6 (3)	222 (97)	2 (1)
Ciprofloxacin	105	7 (7)	94 (90)	4 (4)

^a^Variables have missing data.

### Switching from parenteral to oral antibiotics

Eighty-one percent (491/603; 95% CI: 78%–85%) of patients who used antibiotics during their hospital stay received at least one parenteral formulation of the antibiotic(s). In particular, 77% (188/245; 95% CI: 73%–80%) of patients on metronidazole and 76% (87/114; 95% CI: 73%–80%) on ciprofloxacin received at least one intravenous dose of their prescribed drug (see Table 2 and Table S2). Only 7% (13/188; 95% Poisson CI: 4%–12%) of patients on intravenous metronidazole and 6% (5/87; 95% Poisson CI: 2%–13%) on intravenous ciprofloxacin switched from intravenous to oral antibiotic medication.

### Parenteral/oral antibiotic administration and missed-dose days

Missing at least one dose-day of any of the five frequently used hospital-initiated antibiotics occurred in 46% (222/485; 95% CI: 41%–50%) of inpatients who received at least one parenteral form of antibiotic, but in 29% (21/73; 95% CI: 19%–41%) of those who used the oral route only [χ^2^ (1 df) = 7.46; *P* = 0.006].

### Prescription errors of the three most frequently used hospital-initiated antibiotics

Overall, treatment duration was omitted by the prescriber in 9% (47/536; 95% CI: 7%–11%) of patients who received the oral/intravenous form of ceftriaxone, metronidazole or ciprofloxacin. Treatment duration was omitted by the prescriber in: 7% (27/398; 95% CI: 4%–9%) of patients for whom intravenous ceftriaxone was prescribed; 7% (16/245; 95% CI: 3%–10%) for oral/intravenous metronidazole; and 8% (9/114; 95% Poisson CI: 4%–15%) for oral/intravenous ciprofloxacin.

## Discussion

### Antibiotic prescription and consumption

A high proportion of inpatients used antibiotics in the month preceding hospitalization (39%), predominantly co-trimoxazole and dapsone (18%, 136/762), for prophylaxis against opportunistic infections in HIV-positive patients.^17,20^ A household survey of 2914 cases from five African countries (Gambia, Ghana, Kenya, Nigeria and Uganda) reported a similar proportion (36%) of antibiotic use in the community.^21^ A comparable prevalence of antibiotic use (30%), as measured by the antibacterial activity of the urine samples of 450 outpatients at two regional referral hospitals in northern Uganda, was recently reported.^22^ Antibiotic use may increase during influenza seasons.^23^ Influenza infections occur all year round in Uganda but peak during October—November, which coincides with the second, heavier rainy season of the year that spans September—November,^24^ at the tail-end of which we commenced data collection.

Three-quarters of inpatients received at least one antibiotic during their hospital stay whether co-trimoxazole and dapsone users were included or excluded from the analysis. High rates of hospital-initiated antibiotic use among inpatients are consistently reported in other resource-limited settings: 83% among 435 medical and surgical inpatients in a 60 bed hospital located in a small Indian community; and 79% (*n* = 5381) and 82% (*n* = 2463) among inpatients at 350 bed and 570 bed tertiary health facilities, respectively, in a large Indian community.^25,26^

Although antibiotics were extensively used, our inpatients were frequently underdosed on the prescribed/dispensed antibiotics. The measured extent of antibiotic use in our setting, as quantified by DDDs per 1000 patient-days (531), was similar to Dutch hospitals (523 in 2003 and 698 in 2009), yet Dutch hospitals are known to have the lowest levels of total antibiotic consumption in Europe.^27^ Also, a South African study reported similar DDDs per 1000 patient-days (592) in the pre-intervention arm of a ward-based antibiotic stewardship programme.^28^ If all prescribed doses were administered to inpatients, clearly antibiotic consumption in Uganda, as measured by DDDs per 1000 inpatient-days, would be automatically higher than the Dutch estimates of antibiotic use.

Prescribers might be more certain of the treatment needs of inpatients with single working diagnoses and those with unchanged diagnoses from admission to discharge, which might partly explain why both inpatient groups had the lowest proportions of antibiotic use. The extensive antibiotic prescription/use rates during hospitalization could, in part, be driven by uncertainties in working diagnoses due to the lack of rapid point-of-care tests in our setting.^13^ As reported elsewhere,^3,21,29^ respiratory conditions ranked among the most frequent diagnoses linked to antibiotic prescribing.

### Prescription adherence/missed doses

The per-day risk of missed-dose days was greatest on day 1 of prescribed antibiotic. Also, the receipt of only a single dose-day of antibiotic treatment was common even where a 5 day course was prescribed. Clearly, it is more serious to miss the one daily dose of ceftriaxone, used presumably for severe infections such as meningitis, than a dose of thrice-daily metronidazole. Also, to be effective, ceftriaxone relies on its long *t*_1/2_ and the *T*_>MIC_, while e.g. ciprofloxacin has a concentration-dependent bactericidal effect (AUC/MIC ratio).^30,31^ A multicentre audit of administered antimicrobials in England found that 13% (802/6062) of patients had missed at least one prescribed dose, which is 3-fold less than the proportion reported in our cohort (44%); the main reasons for omitted doses were ‘drugs were not available’, ‘patient refused’, ‘prescribed route was not available’ or ‘patient was away from ward’.^32^ The UK's National Patient Safety Agency has observed that patient harm from omitted doses is mainly by antimicrobials.^33^ Delayed initiation of prescribed antibiotic medication coupled with frequent missed-dose days and the failure to complete full courses of prescribed antibiotic treatment might result in temporary or permanent patient harm from lack of adequate treatment effect.^34,35^ Excess morbidity/mortality for undertreated patients could not be reliably measured in our study. However, missed antibiotic doses can promote the occurrence of life-threatening conditions, such as sepsis, and critically ill patients in septic shock cannot afford to miss their antibiotic treatment.^36^ Exposure of microorganisms to non-lethal subtherapeutic drug concentrations also increases the risk of antibiotic resistance.^37^

### Parenteral/oral switch

A low proportion of patients who received hospital-initiated metronidazole (7%) or ciprofloxacin (6%) switched from intravenous to oral antibiotic medication, suggesting heavy inclination towards parenteral antibiotic administration. The use of parenteral antibiotic formulations was associated with a higher risk of missing at least one antibiotic dose-day, typically the first, which merits further investigation. Patients relied entirely on the nurses to administer parenteral antibiotics. Ward nurses administered parenteral medicines at scheduled times implying that a patient had a higher risk of missing his/her dose(s) if not in bed or did not readily have the prescribed parenteral medication during the nurse's scheduled visit. Oral medications can, however, be self-administered by the patient whenever available without reliance on the ward nurse, thus, possibly lowering the risk of missing a dose-day. See the Supplementary Discussion for further details on drug administration issues. The relationship between missing the first dose-day of antibiotic and route of administration could have been confounded by the higher proportion of parenteral medications usually prescribed at the beginning of hospital stay compared with oral medications largely prescribed towards hospital discharge. Patients should switch from parenteral to oral medication at the earliest opportunity to reduce the risk of patient harm associated with parenteral medications, such as use of the wrong diluent or wrong rate of intravenous medication administration.^38^ The parenteral/oral switch also facilitates discharge from hospital if patients have recovered well, further reducing associated healthcare costs of longer hospital stay.^39^ Multiple interventions, ranging from written guidelines and educational programmes to antimicrobial stewardship,^13,37,40^ may be needed to regulate the use of parenteral antibiotics (and other drug classes) in our resource-limited setting.

### Prescription errors

Treatment duration was omitted by the prescriber in 7%–8% of prescriptions of the frequently administered antibiotics. These prescription errors, mainly by junior doctors, were usually corrected by senior house officers and consultants within 12 h during subsequent ward round(s). Thus, the system has already built a solution to correct staff prescription errors. Prescription errors will often be committed, but a system that checks for and corrects those errors is more robust.

### Organizational issues

Missed/delayed doses in our setting might be attributed to drug stock-outs, understaffing and inadequate communication between HCPs and with the patients, all of which are system-related problems that should be addressed at the organizational level. Elsewhere, first doses compared with subsequent doses are twice as likely to be missed due to unavailability of the drug.^32^ One in four of our patients on azithromycin purchased the medicine from a private community pharmacy, thus underpinning the need for stable stocks of free-of-charge essential antibiotics and other critical medicines that many patients cannot afford to buy. To avoid disruptive drug stock-outs, our hospital and possibly other health facilities in similar resource-poor settings could provide mechanisms for urgent supply of crucial medicines whenever needed.^33^

Encouraging proper ward-level documentation of patients' clinical and medication data promotes clearer communication among staff^41^ within shift and at shift changes and is essential for ensuring that patients promptly receive their prescribed medication, especially if the patient/caregiver needs to fill prescriptions at the pharmacy. Adequate record-keeping also provides auditable primary documents that can generate valuable medication safety data. Medication errors occurred at an unacceptably high rate in patients who received oral azithromycin, most of which were medication administration errors by patients. Errors by patients, particularly those with oral medications, are harder to correct due to the absence, in our hospital setting, of a formal mechanism to verify and rectify them. To reduce the pressure on understaffed wards, it may be necessary to provide training and practical assistance (e.g. buzzers, alarms on mobile phones or chimes as reminders of scheduled dosing) to caregivers on e.g. how to monitor and adhere to a patient's oral medication dosing intervals. Involving patients and caregivers in safer healthcare has been encouraged by Ugandan HCPs who endorsed patient participation in the reporting of medication errors.^42^

### Study limitations

There are several limitations to this study. First, the intensity of data collection and associated staff costs coupled with the need to collect high-quality data limited the number of patients studied to the range 600–800 rather than 1200–1500 as originally envisaged. Our initial intention was to recruit almost 250 patients per ward in five wards (four wards in the national referral and one ward in a regional referral hospital), but the achievement of ∼100 patients per ward has had to suffice. Second, we did not have a measure of antibiotic resistance engendered by curtailed antibiotic courses for patients admitted into the four study wards. Third, we encountered deviations from the random sampling schedule for patients to join the study cohort. However, the deviations from planned recruitment are unlikely to undermine the major finding on underadministration of prescribed antibiotics. Fourth, refusal rates by those omitted were not formally recorded, but were generally low. Fifth, we observed an excess of administered intravenous ceftriaxone doses over dispensed ceftriaxone doses (see Table S5 for possible explanations). Sixth, the study was conducted at a national referral and teaching hospital, which may not be representative of antibiotic prescribing practices at lower-level, particularly peripheral, health facilities in Uganda.

### Conclusions

High rates of antibiotic use both pre-admission and during hospitalization were observed, with low parenteral/oral switch of intravenous metronidazole/ciprofloxacin. Antibiotic underdosing was common and resulted mostly from delayed/missed doses of prescribed/dispensed antibiotics and ultimately from failure to complete full courses of prescribed antibiotic medication. Extensive exposure of patients to antibiotics coupled with underdosing risks loss of efficacy and drug resistance, which could wipe out the limited affordable antibiotic treatment options available in our low-resource setting.

## Funding

R. K. gratefully acknowledges funding support provided by the Training Health Researchers into Vocational Excellence (THRiVE) in East Africa grant number 087540, funded by the Wellcome Trust and an African Doctoral Dissertation Research Fellowship (ADDRF) award 2013-2015 ADF 006 offered by the African Population and Health Research Centre (APHRC) in partnership with the International Development Research Centre (IDRC). In the UK, S. M. B. is funded by Medical Research Council programme number MC_U105260794. The funders had no role in the decisions on what and where to publish.

## Transparency declarations

S. M. B. holds GSK shares. Both other authors: none to declare.

### Author contributions

R. K. conceived of the study and drafted the manuscript and, in conjunction with S. M. B., participated in its design, implementation, statistical analysis and drawing of inferences. C. K. participated in the study design and, together with S. M. B., took part in the manuscript writing process. All authors approved the final manuscript.

## Disclaimer

The work here reported is solely the responsibility of the authors and does not necessarily represent the official views of the supporting offices.

## Supplementary data

Supplementary Methods, Tables S1 to S5, Figure S1, Supplementary Results and Supplementary Discussion are available as Supplementary data at *JAC* Online (http://jac.oxfordjournals.org/).

Supplementary Data
