# Supplementary material for: Extensive antibiotic prescription rate among hospitalized patients in Uganda: but with frequent missed-dose days
Source: J Antimicrob Chemother. 2016 Mar 5;71(6):1697–706. doi: 10.1093/jac/dkw025 (PMC4867101; doi:10.1093/jac/dkw025)
Supplement: Supplementary Data [file supp_71_6_1697__index.html]

Extensive antibiotic prescription rate among hospitalized patients in Uganda: but with frequent missed-dose days — Extensive antibiotic prescription rate among hospitalized patients in Uganda: but with frequent missed-dose days — Supplementary Data 

# Extensive antibiotic prescription rate among hospitalized patients in Uganda: but with frequent missed-dose days

## Supplementary Data

Supplementary Data

- Supplementary Data - Docx file
