# Supplementary material for: Extensive antibiotic prescription rate among hospitalized patients in Uganda: but with frequent missed-dose days
Source: J Antimicrob Chemother. 2016 Mar 5;71(6):1697–706. doi: 10.1093/jac/dkw025 (PMC4867101; doi:10.1093/jac/dkw025)
Supplement: Supplementary Data [file supp_dkw025_dkw025supp.docx]

**Supplementary data**

**Supplementary Methods: Training of data collection teams and further details on data collection/management**

Training of data collection teams

Training encompassed the different phases of drug development, the role of phase IV or pharmacovigilance in patient safety, and of pharmacoepidemiology in quantifying adverse drug reactions (ADRs). The training covered adverse events (AEs); suspected ADRs; medication errors (MEs); and causality assessment of AEs. Inter alia, trainees were instructed on differences between; i) AEs & suspected ADRs; ii) serious and severe suspected ADRs; iii) expected vs. unexpected ADRs (types A & B); iv) rare vs. non-rare suspected ADRs; v) trigger tools as a method for improving diagnosis of suspected ADRs; vi) preventability of ADRs; vii) the Charlson co-morbidity index, and viii) identification of MEs.

Basic concepts of clinical research and how it differs from clinical care were detailed including the principles of informed consent. The major ADRs attributed to different pharmacological drug classes were discussed, not least the different routes of drug administration.

Data collection

In each research team, clinical data were captured from clinical notes in the patient’s file, from clinical examination of the patient by each team’s medical officer, and by patient/caregiver/ward staff interviews. Each research team’s pharmacist interviewed the patient at recruitment or used the patient’s available medical documents to obtain baseline information on medications used in the 4-weeks preceding hospitalization. During the patient’s hospital stay, medication data were obtained from clinical notes, treatment sheets, and drug administration charts in the patient’s hospital file, dispensing records of ward pharmacies, pill count validation of a patient’s oral medication (tablets, capsules) and by viewing of unused injectable medicine vials/ampoules in the possession of the patient/caregiver. Drug information was also obtained by daily interviews with the patient/caregiver or ward staff to elicit information on prescribed, dispensed, and administered medicines for which little or no other evidence might have been available – such as oral medications taken by the patient (validated by pill counts) which, ordinarily, are not recorded by ward staff onto patients’ drug administration charts.

Data management

Additional data included: patient’s serial number (to delink the data from the patient’s identity); prescription data; dispensing data; medicines administration and pharmacist’s medication review data [number of administered medicines, and route of administration (intravenous/intramuscular, IV/IM or otherwise)]; medication error (ME) tracking data; and adverse events tracking data. In particular, an alphabetical coding-frame of all administered medicines was constructed and likewise of all working/final diagnoses for the cohort.

Using Epidata 3.1 software with check programs to limit out-of-range data entry errors, the first 201 cases were entered into a databank by RK to familiarize himself with the data, with the expected data-entry time per case, and with any problems that a data-entry clerk would encounter, while the subsequent 561 cases were entered by a pre-trained data entry clerk.

To verify the quality of data abstraction and entry, 10% of the 762 CRFs (26-page study schedule) were randomly re-sampled using Stata 12.0[^16^](#_ENREF_16) to generate 76 cases for whom previously abstracted data were re-abstracted by RK and re-entered into the Epidata 3.1 databank by the pre-trained data entry clerk. Double-data entry verification was used to identify and record discrepancies between previously entered data against data capture at the re-entry phase. Where data discrepancies occurred, the original CRF was cross-checked and the correct entries were made. Where the estimated discrepancy rate did not exclude a 10% data entry error-rate as the upper 95% confidence limit, RK re-checked all 762 original records for all important such fields. A list of fields for which 10% error-rate could not be ruled out initially is outlined below:

- Number of dispensed medicines for which labeling was assessed
- Number of dispensed medicines for which patient care was assessed
- Number of administered medicines
- Number of medication errors
- Number of stage four medication errors
- Number of possible suspected adverse drug reactions
- Number of doses for the most frequently prescribed antibiotics

| **Table S1. Computation of Defined Daily Doses (DDDs), DDDs per 1000 patient-days, and DDDs per 100 hospital admissions for 762 hospitalized patients, Uganda, 2014** | | | | | | |
| --- | --- | --- | --- | --- | --- | --- |
| **Description** | **Number of doses administered** | **Doses administered (g or tablets)** | **WHO DDD (mg, g or tab)** | **DDDs administered** | **DDD/1000 Patient-days^±^** | **DDD/100 Hospital admissions*** |
| **Cephalosporins J01D** |  |  |  |  |  |  |
| Ceftriaxone 2g IV (J01DD04) | 398 | 796 g | 2 g | 398.0 | 106.4 | 52.2 |
| Cefixime 200mg po (J01DD08) | 17 | 3.4 g | 0.4 g | 8.5 | 2.3 | 1.1 |
| Cefuroxime 500mg po (J01DC02) | 2 | 1 g | 0.5 g | 2.0 | 0.5 | 0.3 |
| Cefotaxime 2g IV (J01DD01) | 2 | 4 g | 4 g | 1.0 | 0.3 | 0.1 |
| **Subtotal** |  |  |  |  | **109.5** | **53.7** |
|  |  |  |  |  |  |  |
| **Combinations of Sulphonamides with** |  |  |  |  |  |  |
| **Trimethoprim J01EE** |  |  |  |  |  |  |
| Co-trimoxazole 480mg po (J01EE01) | 1,432 | 1432 tabs | 4 tabs | 358.0 | **95.7** | **47.0** |
|  |  |  |  |  |  |  |
| **Fluoroquinolones J01MA** |  |  |  |  |  |  |
| Ciprofloxacin 500mg po (J01MA02) | 123 | 61.5 g | 1 g | 61.5 | 16.4 | 8.1 |
| Ciprofloxacin 400mg IV (J01MA02) | 273 | 109.2 g | 0.5 g | 218.4 | 58.4 | 28.7 |
| Levofloxacin 500mg po (J01MA12) | 15 | 7.5 g | 0.5 g | 15.0 | 4.0 | 2.0 |
| Levofloxacin 500mg IV (J01MA12) | 47 | 23.5 g | 0.5 g | 47.0 | 12.6 | 6.2 |
| Moxifloxacin 400mg po (J01MA14) | 1 | 0.4 g | 0.4 g | 1.0 | 0.3 | 0.1 |
| **Subtotal** |  |  |  |  | **91.7** | **45.0** |
|  |  |  |  |  |  |  |
| **Imidazole derivatives J01XD*** |  |  |  |  |  |  |
| Metronidazole 400mg po (P01AB01 ) | 289 | 115.6 g | 2 g | 57.8 | 15.5 | 7.6 |
| Metronidazole 500mg IV (J01XD01) | 754 | 377 g | 1.5 g | 251.3 | 67.2 | 33.0 |
| Tinidazole 500mg po (P01AB02) | 9 | 4.5 g | 2 g | 2.3 | 0.6 | 0.3 |
| **Subtotal** |  |  |  |  | **83.3** | **40.9** |
|  |  |  |  |  |  |  |
| **Macrolides, lincosamides and streptogramins J01F** |  |  |  |  |  |  |
| Azithromycin 500mg po (J01FA10) | 74 | 37 g | 0.3 g | 123.3 | 33.0 | 16.2 |
| Erythromycin 500mg po (J01FA01) | 159 | 79.5 g | 1 g | 79.5 | 21.3 | 10.4 |
| Clarithromycin 500mg po (J01FA09) | 44 | 22 g | 0.5 g | 44.0 | 11.8 | 5.8 |
| Clindamycin 600mg IV (J01FF01) | 5 | 3 g | 1.8 g | 1.7 | 0.4 | 0.2 |
| **Subtotal** |  |  |  |  | **66.5** | **32.6** |
|  |  |  |  |  |  |  |
| **Penicillins J01C** |  |  |  |  |  |  |
| Amoxicillin 500mg po (J01CA04) | 338 | 169 g | 1 g | 169.0 | 45.2 | 22.2 |
| Amoxil/Clavulanate 625mg po (J01CR02) | 30.4 | 19 g | 1 g | 19.0 | 5.1 | 2.5 |
| Ampiclox 1g IV (J01CR50) | 25.0 | 25 g | 2 g | 12.5 | 3.3 | 1.6 |
| Ampiclox 500mg po (J01CR50) | 95 | 47.5 tabs | 4 tabs | 11.9 | 3.2 | 1.6 |
| Ampicillin 1g IV (J01CA01) | 17.5 | 17.5 g | 2 g | 8.8 | 2.3 | 1.1 |
| Cloxacillin 500mg IV (J01CF02) | 22 | 11 g | 2 g | 5.5 | 1.5 | 0.7 |
| Benzylpenicillin 2.4MU IV [2.4*0.6] (J01CE08) | 2 | 2.88 g | 3.6 g | 0.8 | 0.2 | 0.1 |
| **Subtotal** |  |  |  |  | **60.8** | **29.8** |
|  |  |  |  |  |  |  |
| **Antimycobacterials J04B** |  |  |  |  |  |  |
| Dapsone 100mg po (J04BA02) | 28 | 2800 mg | 50 mg | 56.0 | 15.0 | 7.3 |
|  |  |  |  |  |  |  |
| **Aminoglycosides J01G** |  |  |  |  |  |  |
| Gentamycin 160 mg IV (J01GB03) | 22.5 | 3.6 g | 0.24 g | 15.0 | 4.0 | 2.0 |
|  |  |  |  |  |  |  |
| **Tetracyclines J01A** |  |  |  |  |  |  |
| Doxycycline 100mg po (J01AA02) | 16 | 1.6 g | 0.1 g | 16.0 | 4.3 | 2.1 |
|  |  |  |  |  |  |  |
| **Carbapenems J01DH** |  |  |  |  |  |  |
| Meropenem 500mg IV (JO1DH02) | 1 | 0.5 g | 2 g | 0.3 | 0.1 | 0.0 |
|  |  |  |  |  |  |  |
| **Total DDDs/Total DDDs per 1000 Patient-days/Total DDDs per 100 Hospital admissions** | | | | **1,985.0** | **530.9** | **260.5** |
| ^±^A total of 3,741 in-hospital patient-days were experienced; *Each of the 762 inpatients were admitted once into the hospital during the study period | | | | | | |

| **Table S2. Patterns of systemic antibiotic use among 762 hospitalized patients, Uganda, 2014** | | | | | | | | | | | | | | | |
| --- | --- | --- | --- | --- | --- | --- | --- | --- | --- | --- | --- | --- | --- | --- | --- |
| **Antibiotic name** | | **Oral** | **IV/IM** | | **No. of patients** | | **Patterns of systemic antibiotic use** | | | | | | | | |
|  |  |  |  |  |  |  | **Defined Daily Doses (DDDs)** | | | **% DDDs** | | **DDDs/1000 patient-days** | | | **DDDs/100 admissions** |
| **Cephalosporins J01D** | |  |  | |  | |  | | |  | |  | | |  |
| Ceftriaxone (J01DD04) | | 0 | 398 | | 398 | | 398.0 | | | 20 | | 106.4 | | | 52.2 |
| Others | | 5 | 0 | | 5 | | 11.5 | | |  | |  | | | 1.5 |
| Cefixime (J01DD08) | | 2 | 0 | | 2 | | 8.5 | | |  | | 2.3 | | | 1.1 |
| Cefuroxime (J01DC02) | | 2 | 0 | | 2 | | 2.0 | | |  | | 0.5 | | | 0.3 |
| Cefotaxime (J01DD01) | | 1 | 0 | | 1 | | 1.0 | | |  | | 0.3 | | | 0.1 |
| **Total** | | **5** | **398** | | **403** | | **409.5** | | | **21** | | **109.5** | | | **53.7** |
|  | |  |  | |  | |  | | |  | |  | | |  |
| **Combinations of Sulphonamides with** | | | | |  | |  | | |  | |  | | |  |
| **Trimethoprim J01EE** | |  |  | |  | |  | | |  | |  | | |  |
| Co-trimoxazole (J01EE01) | | 162 | 0 | | 162 | | 358.0 | | | 18 | | 95.7 | | | 47.0 |
| **Total** | | **162** | **0** | | **162** | | **358.0** | | | **18** | | **95.7** | | | **47.0** |
|  | |  |  | |  | |  | | |  | |  | | |  |
| **Fluoroquinolones J01MA** | |  |  | |  | |  | | |  | |  | | |  |
| Ciprofloxacin (J01MA02) | | 27 | 87 | | 114 | | 279.9 | | | 14 | | 74.9 | | | 36.7 |
| Levofloxacin (J01MA12) | | 5 | 12 | | 17 | | 62.0 | | |  | | 16.6 | | | 8.1 |
| Moxifloxacin (J01MA14) | | 1 | 0 | | 1 | | 1.0 | | |  | | 0.3 | | | 0.1 |
| **Total** | | **33** | **99** | | **132** | | **342.9** | | | **17** | | **91.7** | | | **45.0** |
|  | |  |  | |  | |  | | |  | |  | | |  |
| **Imidazole derivatives J01XD**^e^ | |  |  | |  | |  | | |  | |  | | |  |
| Metronidazole (J01XD01/P01AB01)^g^ | | 58 | 188 | | 246 | | 309.1 | | | 16 | | 82.7 | | | 40.6 |
| Tinidazole (P01AB02) | | 2 | 0 | | 2 | | 2.3 | | |  | | 0.6 | | | 0.3 |
| **Total** | | **60** | **188** | | **248** | | **311.4** | | | **16** | | **83.3** | | | **40.9** |
|  | |  |  | |  | |  | | |  | |  | | |  |
| **Macrolides, lincosamides and streptogramins J01F** | |  |  | |  | |  | | |  | |  | | |  |
| Azithromycin (J01FA10) | | 26 | 0 | | 26 | | 123.3 | | | 6 | | 33.0 | | | 16.2 |
| Erythromycin (J01FA01) | | 19 | 0 | | 19 | | 79.5 | | |  | | 21.3 | | | 10.4 |
| Clarithromycin (J01FA09) | | 11 | 0 | | 11 | | 44.0 | | |  | | 11.8 | | | 5.8 |
| Clindamycin (J01FF01) | | 0 | 1 | | 1 | | 1.7 | | |  | | 0.4 | | | 0.2 |
| **Total** | | **56** | **1** | | **57** | | **248.5** | | | **13** | | **66.5** | | | **32.6** |
|  | |  |  | |  | |  | | |  | |  | | |  |
| **Penicillins J01C** | |  |  | |  | |  | | |  | |  | | |  |
| Amoxicillin (J01CA04) | | 57 | 0 | | 57 | | 169.0 | | | 9 | | 45.2 | | | 22.2 |
| Amoxicillin+clavulanic acid (J01CR02) | | 8 | 0 | | 8 | | 19.0 | | |  | | 5.1 | | | 2.5 |
| Ampicillin (J01CA01) | | 0 | 11 | | 11 | | 8.8 | | |  | | 2.3 | | | 1.1 |
| Ampicillin+cloxacillin (J01CR50) | | 19 | 13 | | 32 | | 24.4 | | |  | | 6.5 | | | 3.2 |
| Cloxacillin (J01CF02) | | 0 | 2 | | 2 | | 5.5 | | |  | | 1.5 | | | 0.7 |
| Benzylpenicillin (J01CE08) | | 0 | 2 | | 2 | | 0.8 | | |  | | 0.2 | | | 0.1 |
| **Total** | | **84** | **28** | | **112** | | **227.4** | | | **11** | | **60.8** | | | **29.8** |
|  | |  |  | |  | |  | | |  | |  | | |  |
| **Antimycobacterials J04B** | |  |  | |  | |  | | |  | |  | | |  |
| Dapsone (J04BA02) | | 5 | 0 | | 5 | | 56.0 | | |  | | 15.0 | | | 7.3 |
| **Total** | | **5** | **0** | | **5** | | **56.0** | | | **3** | | **15.0** | | | **7.3** |
|  | |  |  | |  | |  | | |  | |  | | |  |
| **Aminoglycosides J01G** | |  |  | |  | |  | | |  | |  | | |  |
| Gentamicin (J01GB03) | | 0 | 11 | | 11 | | 15.0 | | |  | | 4.0 | | | 2.0 |
| **Total** | | **0** | **11** | | **11** | | **15.0** | | | **1** | | **4.0** | | | **2.0** |
|  | |  |  | |  | |  | | |  | |  | | |  |
| **Tetracyclines J01A** | |  |  | |  | |  | | |  | |  | | |  |
| Doxycycline (J01AA02) | | 5 | 0 | | 5 | | 16.0 | | |  | | 4.3 | | | 2.1 |
| **Total** | | **5** | **0** | | **5** | | **16.0** | | | **1** | | **4.3** | | | **2.1** |
|  | |  |  | |  | |  | | |  | |  | | |  |
| **Carbapenems J01DH** | |  |  | |  | |  | | |  | |  | | |  |
| Meropenem (JO1DH02) | | 0 | 1 | | 1 | | 0.3 | | |  | | 0.1 | | | 0.0 |
| **Total** | | **0** | **1** | | **1** | | **0.3** | | | **0** | | **0.1** | | | 0.0 |
|  | |  |  | |  | |  | | |  | |  | | |  |
| **Overall Total** | | | | | | | **1985.0** | | |  | | **530.9** | | | **260.5** |
|  | | | |  | |  |  |  | | | | |  | | |
| **Route of antibiotic administration** | | | | | | | | |  | |  | | |  | |
| Oral (po) |  | |  | | 112 | | 1024.8 | | 52 | | 274.1 | | | **134.5** | |
| Parenteral (IV/IM) |  | |  | | 491 | | 960.2 | | 48 | | 256.8 | | | **126.0** | |
| **Total** |  | |  | | **603** | | **1985.0** | | **100** | | **530.9** | | | **260.5** | |
| ^e^Parenteral formulations of nitroimidazoles are classified as J01XD and oral formulations as P01AB in the WHO ATC/DDD index;  ^g^Overall, 246 patients received metronidazole but one patient did not have details | | | | | | | | | | | | | | | |

| **Table S3. Missed dose-days of the five most frequently used hospital-initiated antibiotics (ceftriaxone, metronidazole, ciprofloxacin, amoxicillin, and azithromycin), Uganda, 2014** | | | | | | | | | | | | | | | | | | | | | | | | | | | |  | | | | | | | |  | |  | | | | | | |  | | | |  |  |
| --- | --- | --- | --- | --- | --- | --- | --- | --- | --- | --- | --- | --- | --- | --- | --- | --- | --- | --- | --- | --- | --- | --- | --- | --- | --- | --- | --- | --- | --- | --- | --- | --- | --- | --- | --- | --- | --- | --- | --- | --- | --- | --- | --- | --- | --- | --- | --- | --- | --- | --- |
| **Missed dose-days of ceftriaxone injection among 398 hospitalized patients who received in-hospital intravenous ceftriaxone, Uganda, 2014** | | | | | | | | | | | | | | | | | | | | | | | | | | | |  | | | |  | | | | | |  | | | | | | |  | | | |  |  |
| **Length of stay, days** | **Days missed** | | **Day 1** | **Day 2** | | **Day 3** | | **Day 4** | | **Day 5** | | | **Day 6** | | **Day 7** | | **Day 8** | | | | **Day 9** | | | **Day 10** | | | |  | | | |  | | | | | |  | | | | | | |  | | | |  |  |
| 2 | 1 | |  |  | |  | |  | |  | | |  | |  | |  | | | |  | | |  | | | |  | | | |  | | | | | |  | | | | | | |  | | | |  |  |
| 2 | 1 | |  |  | |  | |  | |  | | |  | |  | |  | | | |  | | |  | | | |  | | | |  | | | | | |  | | | | | | |  | | | |  |  |
| 2 | 1 | |  |  | |  | |  | |  | | |  | |  | |  | | | |  | | |  | | | |  | | | |  | | | | | |  | | | | | | |  | | | |  |  |
| 2 | 1 | |  |  | |  | |  | |  | | |  | |  | |  | | | |  | | |  | | | |  | | | |  | | | | | |  | | | | | | |  | | | |  |  |
| 2 | 1 | |  |  | |  | |  | |  | | |  | |  | |  | | | |  | | |  | | | |  | | | | **KEY** | | | | | |  | | | | | | |  | | | |  |  |
| 2 | 1 | |  |  | |  | |  | |  | | |  | |  | |  | | | |  | | |  | | | |  | | | |  | | | | | | Dose missed | | | | | | | | | | |  |  |
| 2 | 1 | |  |  | |  | |  | |  | | |  | |  | |  | | | |  | | |  | | | |  | | | |  | | | | | | Dose received | | | | | | | | | | |  |  |
| 2 | 1 | |  |  | |  | |  | |  | | |  | |  | |  | | | |  | | |  | | | |  | | | |  | | | | | |  | | | | | | |  | | | |  |  |
| 2 | 1 | |  |  | |  | |  | |  | | |  | |  | |  | | | |  | | |  | | | |  | | | |  | | | | | |  | | | | | | |  | | | |  |  |
| 2 | 1 | |  |  | |  | |  | |  | | |  | |  | |  | | | |  | | |  | | | |  | | | |  | | | | | |  | | | | | | |  | | | |  |  |
| 2 | 1 | |  |  | |  | |  | |  | | |  | |  | |  | | | |  | | |  | | | |  | | | |  | | | | | |  | | | | | | |  | | | |  |  |
| 2 | 1 | |  |  | |  | |  | |  | | |  | |  | |  | | | |  | | |  | | | |  | | | |  | | | | | |  | | | | | | |  | | | |  |  |
| 2 | 1 | |  |  | |  | |  | |  | | |  | |  | |  | | | |  | | |  | | | |  | | | |  | | | | | |  | | | | | | |  | | | |  |  |
| 3 | 1 | |  |  | |  | |  | |  | | |  | |  | |  | | | |  | | |  | | | |  | | | |  | | | | | |  | | | | | | |  | | | |  |  |
| 3 | 1 | |  |  | |  | |  | |  | | |  | |  | |  | | | |  | | |  | | | |  | | | |  | | | | | |  | | | | | | |  | | | |  |  |
| 3 | 1 | |  |  | |  | |  | |  | | |  | |  | |  | | | |  | | |  | | | |  | | | |  | | | | | |  | | | | | | |  | | | |  |  |
| 3 | 1 | |  |  | |  | |  | |  | | |  | |  | |  | | | |  | | |  | | | |  | | | |  | | | | | |  | | | | | | |  | | | |  |  |
| 3 | 1 | |  |  | |  | |  | |  | | |  | |  | |  | | | |  | | |  | | | |  | | | |  | | | | | |  | | | | | | |  | | | |  |  |
| 3 | 1 | |  |  | |  | |  | |  | | |  | |  | |  | | | |  | | |  | | | |  | | | |  | | | | | |  | | | | | | |  | | | |  |  |
| 3 | 1 | |  |  | |  | |  | |  | | |  | |  | |  | | | |  | | |  | | | |  | | | |  | | | | | |  | | | | | | |  | | | |  |  |
| 3 | 1 | |  |  | |  | |  | |  | | |  | |  | |  | | | |  | | |  | | | |  | | | |  | | | | | |  | | | | | | |  | | | |  |  |
| 3 | 1 | |  |  | |  | |  | |  | | |  | |  | |  | | | |  | | |  | | | |  | | | |  | | | | | |  | | | | | | |  | | | |  |  |
| 3 | 1 | |  |  | |  | |  | |  | | |  | |  | |  | | | |  | | |  | | | |  | | | |  | | | | | |  | | | | | | |  | | | |  |  |
| 3 | 1 | |  |  | |  | |  | |  | | |  | |  | |  | | | |  | | |  | | | |  | | | |  | | | | | |  | | | | | | |  | | | |  |  |
| 3 | 1 | |  |  | |  | |  | |  | | |  | |  | |  | | | |  | | |  | | | |  | | | |  | | | | | |  | | | | | | |  | | | |  |  |
| 3 | 1 | |  |  | |  | |  | |  | | |  | |  | |  | | | |  | | |  | | | |  | | | |  | | | | | |  | | | | | | |  | | | |  |  |
| 3 | 1 | |  |  | |  | |  | |  | | |  | |  | |  | | | |  | | |  | | | |  | | | |  | | | | | |  | | | | | | |  | | | |  |  |
| 3 | 1 | |  |  | |  | |  | |  | | |  | |  | |  | | | |  | | |  | | | |  | | | |  | | | | | |  | | | | | | |  | | | |  |  |
| 3 | 1 | |  |  | |  | |  | |  | | |  | |  | |  | | | |  | | |  | | | |  | | | |  | | | | | |  | | | | | | |  | | | |  |  |
| 4 | 3 | |  |  | |  | |  | |  | | |  | |  | |  | | | |  | | |  | | | |  | | | |  | | | | | |  | | | | | | |  | | | |  |  |
| 4 | 2 | |  |  | |  | |  | |  | | |  | |  | |  | | | |  | | |  | | | |  | | | |  | | | | | |  | | | | | | |  | | | |  |  |
| 4 | 2 | |  |  | |  | |  | |  | | |  | |  | |  | | | |  | | |  | | | |  | | | |  | | | | | |  | | | | | | |  | | | |  |  |
| 4 | 1 | |  |  | |  | |  | |  | | |  | |  | |  | | | |  | | |  | | | |  | | | |  | | | | | |  | | | | | | |  | | | |  |  |
| 4 | 1 | |  |  | |  | |  | |  | | |  | |  | |  | | | |  | | |  | | | |  | | | |  | | | | | |  | | | | | | |  | | | |  |  |
| 4 | 1 | |  |  | |  | |  | |  | | |  | |  | |  | | | |  | | |  | | | |  | | | |  | | | | | |  | | | | | | |  | | | |  |  |
| 4 | 1 | |  |  | |  | |  | |  | | |  | |  | |  | | | |  | | |  | | | |  | | | |  | | | | | |  | | | | | | |  | | | |  |  |
| 4 | 1 | |  |  | |  | |  | |  | | |  | |  | |  | | | |  | | |  | | | |  | | | |  | | | | | |  | | | | | | |  | | | |  |  |
| 4 | 1 | |  |  | |  | |  | |  | | |  | |  | |  | | | |  | | |  | | | |  | | | |  | | | | | |  | | | | | | |  | | | |  |  |
| 4 | 1 | |  |  | |  | |  | |  | | |  | |  | |  | | | |  | | |  | | | |  | | | |  | | | | | |  | | | | | | |  | | | |  |  |
| 4 | 1 | |  |  | |  | |  | |  | | |  | |  | |  | | | |  | | |  | | | |  | | | |  | | | | | |  | | | | | | |  | | | |  |  |
| 4 | 1 | |  |  | |  | |  | |  | | |  | |  | |  | | | |  | | |  | | | |  | | | |  | | | | | |  | | | | | | |  | | | |  |  |
| 4 | 1 | |  |  | |  | |  | |  | | |  | |  | |  | | | |  | | |  | | | |  | | | |  | | | | | |  | | | | | | |  | | | |  |  |
| 4 | 1 | |  |  | |  | |  | |  | | |  | |  | |  | | | |  | | |  | | | |  | | | |  | | | | | |  | | | | | | |  | | | |  |  |
| 4 | 1 | |  |  | |  | |  | |  | | |  | |  | |  | | | |  | | |  | | | |  | | | |  | | | | | |  | | | | | | |  | | | |  |  |
| 4 | 1 | |  |  | |  | |  | |  | | |  | |  | |  | | | |  | | |  | | | |  | | | |  | | | | | |  | | | | | | |  | | | |  |  |
| 4 | 1 | |  |  | |  | |  | |  | | |  | |  | |  | | | |  | | |  | | | |  | | | |  | | | | | |  | | | | | | |  | | | |  |  |
| 4 | 1 | |  |  | |  | |  | |  | | |  | |  | |  | | | |  | | |  | | | |  | | | |  | | | | | |  | | | | | | |  | | | |  |  |
| 4 | 1 | |  |  | |  | |  | |  | | |  | |  | |  | | | |  | | |  | | | |  | | | |  | | | | | |  | | | | | | |  | | | |  |  |
| 4 | 1 | |  |  | |  | |  | |  | | |  | |  | |  | | | |  | | |  | | | |  | | | |  | | | | | |  | | | | | | |  | | | |  |  |
| 4 | 1 | |  |  | |  | |  | |  | | |  | |  | |  | | | |  | | |  | | | |  | | | |  | | | | | |  | | | | | | |  | | | |  |  |
| 4 | 1 | |  |  | |  | |  | |  | | |  | |  | |  | | | |  | | |  | | | |  | | | |  | | | | | |  | | | | | | |  | | | |  |  |
| 4 | 1 | |  |  | |  | |  | |  | | |  | |  | |  | | | |  | | |  | | | |  | | | |  | | | | | |  | | | | | | |  | | | |  |  |
| 4 | 1 | |  |  | |  | |  | |  | | |  | |  | |  | | | |  | | |  | | | |  | | | |  | | | | | |  | | | | | | |  | | | |  |  |
| 4 | 1 | |  |  | |  | |  | |  | | |  | |  | |  | | | |  | | |  | | | |  | | | |  | | | | | |  | | | | | | |  | | | |  |  |
| 4 | 1 | |  |  | |  | |  | |  | | |  | |  | |  | | | |  | | |  | | | |  | | | |  | | | | | |  | | | | | | |  | | | |  |  |
| 5 | 2 | |  |  | |  | |  | |  | | |  | |  | |  | | | |  | | |  | | | |  | | | |  | | | | | |  | | | | | | |  | | | |  |  |
| 5 | 2 | |  |  | |  | |  | |  | | |  | |  | |  | | | |  | | |  | | | |  | | | |  | | | | | |  | | | | | | |  | | | |  |  |
| 5 | 2 | |  |  | |  | |  | |  | | |  | |  | |  | | | |  | | |  | | | |  | | | |  | | | | | |  | | | | | | |  | | | |  |  |
| 5 | 2 | |  |  | |  | |  | |  | | |  | |  | |  | | | |  | | |  | | | |  | | | |  | | | | | |  | | | | | | |  | | | |  |  |
| 5 | 2 | |  |  | |  | |  | |  | | |  | |  | |  | | | |  | | |  | | | |  | | | |  | | | | | |  | | | | | | |  | | | |  |  |
| 5 | 2 | |  |  | |  | |  | |  | | |  | |  | |  | | | |  | | |  | | | |  | | | |  | | | | | |  | | | | | | |  | | | |  |  |
| 5 | 2 | |  |  | |  | |  | |  | | |  | |  | |  | | | |  | | |  | | | |  | | | |  | | | | | |  | | | | | | |  | | | |  |  |
| 5 | 1 | |  |  | |  | |  | |  | | |  | |  | |  | | | |  | | |  | | | |  | | | |  | | | | | |  | | | | | | |  | | | |  |  |
| 5 | 1 | |  |  | |  | |  | |  | | |  | |  | |  | | | |  | | |  | | | |  | | | |  | | | | | |  | | | | | | |  | | | |  |  |
| 5 | 1 | |  |  | |  | |  | |  | | |  | |  | |  | | | |  | | |  | | | |  | | | |  | | | | | |  | | | | | | |  | | | |  |  |
| 5 | 1 | |  |  | |  | |  | |  | | |  | |  | |  | | | |  | | |  | | | |  | | | |  | | | | | |  | | | | | | |  | | | |  |  |
| 5 | 1 | |  |  | |  | |  | |  | | |  | |  | |  | | | |  | | |  | | | |  | | | |  | | | | | |  | | | | | | |  | | | |  |  |
| 5 | 1 | |  |  | |  | |  | |  | | |  | |  | |  | | | |  | | |  | | | |  | | | |  | | | | | |  | | | | | | |  | | | |  |  |
| 5 | 1 | |  |  | |  | |  | |  | | |  | |  | |  | | | |  | | |  | | | |  | | | |  | | | | | |  | | | | | | |  | | | |  |  |
| 5 | 1 | |  |  | |  | |  | |  | | |  | |  | |  | | | |  | | |  | | | |  | | | |  | | | | | |  | | | | | | |  | | | |  |  |
| 5 | 1 | |  |  | |  | |  | |  | | |  | |  | |  | | | |  | | |  | | | |  | | | |  | | | | | |  | | | | | | |  | | | |  |  |
| 5 | 1 | |  |  | |  | |  | |  | | |  | |  | |  | | | |  | | |  | | | |  | | | |  | | | | | |  | | | | | | |  | | | |  |  |
| 5 | 1 | |  |  | |  | |  | |  | | |  | |  | |  | | | |  | | |  | | | |  | | | |  | | | | | |  | | | | | | |  | | | |  |  |
| 5 | 1 | |  |  | |  | |  | |  | | |  | |  | |  | | | |  | | |  | | | |  | | | |  | | | | | |  | | | | | | |  | | | |  |  |
| 5 | 1 | |  |  | |  | |  | |  | | |  | |  | |  | | | |  | | |  | | | |  | | | |  | | | | | |  | | | | | | |  | | | |  |  |
| 5 | 1 | |  |  | |  | |  | |  | | |  | |  | |  | | | |  | | |  | | | |  | | | |  | | | | | |  | | | | | | |  | | | |  |  |
| 5 | 1 | |  |  | |  | |  | |  | | |  | |  | |  | | | |  | | |  | | | |  | | | |  | | | | | |  | | | | | | |  | | | |  |  |
| 5 | 1 | |  |  | |  | |  | |  | | |  | |  | |  | | | |  | | |  | | | |  | | | |  | | | | | |  | | | | | | |  | | | |  |  |
| 5 | 1 | |  |  | |  | |  | |  | | |  | |  | |  | | | |  | | |  | | | |  | | | |  | | | | | |  | | | | | | |  | | | |  |  |
| 6 | 4 | |  |  | |  | |  | |  | | |  | |  | |  | | | |  | | |  | | | |  | | | |  | | | | | |  | | | | | | |  | | | |  |  |
| 6 | 3 | |  |  | |  | |  | |  | | |  | |  | |  | | | |  | | |  | | | |  | | | |  | | | | | |  | | | | | | |  | | | |  |  |
| 6 | 3 | |  |  | |  | |  | |  | | |  | |  | |  | | | |  | | |  | | | |  | | | |  | | | | | |  | | | | | | |  | | | |  |  |
| 6 | 2 | |  |  | |  | |  | |  | | |  | |  | |  | | | |  | | |  | | | |  | | | |  | | | | | |  | | | | | | |  | | | |  |  |
| 6 | 2 | |  |  | |  | |  | |  | | |  | |  | |  | | | |  | | |  | | | |  | | | |  | | | | | |  | | | | | | |  | | | |  |  |
| 6 | 2 | |  |  | |  | |  | |  | | |  | |  | |  | | | |  | | |  | | | |  | | | |  | | | | | |  | | | | | | |  | | | |  |  |
| 6 | 2 | |  |  | |  | |  | |  | | |  | |  | |  | | | |  | | |  | | | |  | | | |  | | | | | |  | | | | | | |  | | | |  |  |
| 6 | 1 | |  |  | |  | |  | |  | | |  | |  | |  | | | |  | | |  | | | |  | | | |  | | | | | |  | | | | | | |  | | | |  |  |
| **6** | 1 | |  |  | |  | |  | |  | | |  | |  | |  | | | |  | | |  | | | |  | | | |  | | | | | |  | | | | | | |  | | | |  |  |
| 6 | 1 | |  |  | |  | |  | |  | | |  | |  | |  | | | |  | | |  | | | |  | | | |  | | | | | |  | | | | | | |  | | | |  |  |
| 6 | 1 | |  |  | |  | |  | |  | | |  | |  | |  | | | |  | | |  | | | |  | | | |  | | | | | |  | | | | | | |  | | | |  |  |
| 6 | 1 | |  |  | |  | |  | |  | | |  | |  | |  | | | |  | | |  | | | |  | | | |  | | | | | |  | | | | | | |  | | | |  |  |
| 6 | 1 | |  |  | |  | |  | |  | | |  | |  | |  | | | |  | | |  | | | |  | | | |  | | | | | |  | | | | | | |  | | | |  |  |
| 6 | 1 | |  |  | |  | |  | |  | | |  | |  | |  | | | |  | | |  | | | |  | | | |  | | | | | |  | | | | | | |  | | | |  |  |
| 6 | 1 | |  |  | |  | |  | |  | | |  | |  | |  | | | |  | | |  | | | |  | | | |  | | | | | |  | | | | | | |  | | | |  |  |
| 6 | 1 | |  |  | |  | |  | |  | | |  | |  | |  | | | |  | | |  | | | |  | | | |  | | | | | |  | | | | | | |  | | | |  |  |
| 6 | 1 | |  |  | |  | |  | |  | | |  | |  | |  | | | |  | | |  | | | |  | | | |  | | | | | |  | | | | | | |  | | | |  |  |
| 6 | 1 | |  |  | |  | |  | |  | | |  | |  | |  | | | |  | | |  | | | |  | | | |  | | | | | |  | | | | | | |  | | | |  |  |
| 6 | 1 | |  |  | |  | |  | |  | | |  | |  | |  | | | |  | | |  | | | |  | | | |  | | | | | |  | | | | | | |  | | | |  |  |
| 6 | 1 | |  |  | |  | |  | |  | | |  | |  | |  | | | |  | | |  | | | |  | | | |  | | | | | |  | | | | | | |  | | | |  |  |
| 6 | 1 | |  |  | |  | |  | |  | | |  | |  | |  | | | |  | | |  | | | |  | | | |  | | | | | |  | | | | | | |  | | | |  |  |
| 6 | 1 | |  |  | |  | |  | |  | | |  | |  | |  | | | |  | | |  | | | |  | | | |  | | | | | |  | | | | | | |  | | | |  |  |
| 7 | 3 | |  |  | |  | |  | |  | | |  | |  | |  | | | |  | | |  | | | |  | | | |  | | | | | |  | | | | | | |  | | | |  |  |
| 7 | 3 | |  |  | |  | |  | |  | | |  | |  | |  | | | |  | | |  | | | |  | | | |  | | | | | |  | | | | | | |  | | | |  |  |
| 7 | 2 | |  |  | |  | |  | |  | | |  | |  | |  | | | |  | | |  | | | |  | | | |  | | | | | |  | | | | | | |  | | | |  |  |
| 7 | 1 | |  |  | |  | |  | |  | | |  | |  | |  | | | |  | | |  | | | |  | | | |  | | | | | |  | | | | | | |  | | | |  |  |
| 7 | 1 | |  |  | |  | |  | |  | | |  | |  | |  | | | |  | | |  | | | |  | | | |  | | | | | |  | | | | | | |  | | | |  |  |
| 7 | 1 | |  |  | |  | |  | |  | | |  | |  | |  | | | |  | | |  | | | |  | | | |  | | | | | |  | | | | | | |  | | | |  |  |
| 7 | 1 | |  |  | |  | |  | |  | | |  | |  | |  | | | |  | | |  | | | |  | | | |  | | | | | |  | | | | | | |  | | | |  |  |
| 7 | 1 | |  |  | |  | |  | |  | | |  | |  | |  | | | |  | | |  | | | |  | | | |  | | | | | |  | | | | | | |  | | | |  |  |
| 7 | 1 | |  |  | |  | |  | |  | | |  | |  | |  | | | |  | | |  | | | |  | | | |  | | | | | |  | | | | | | |  | | | |  |  |
| 7 | 1 | |  |  | |  | |  | |  | | |  | |  | |  | | | |  | | |  | | | |  | | | |  | | | | | |  | | | | | | |  | | | |  |  |
| 7 | 1 | |  |  | |  | |  | |  | | |  | |  | |  | | | |  | | |  | | | |  | | | |  | | | | | |  | | | | | | |  | | | |  |  |
| 7 | 1 | |  |  | |  | |  | |  | | |  | |  | |  | | | |  | | |  | | | |  | | | |  | | | | | |  | | | | | | |  | | | |  |  |
| 7 | 1 | |  |  | |  | |  | |  | | |  | |  | |  | | | |  | | |  | | | |  | | | |  | | | | | |  | | | | | | |  | | | |  |  |
| 7 | 1 | |  |  | |  | |  | |  | | |  | |  | |  | | | |  | | |  | | | |  | | | |  | | | | | |  | | | | | | |  | | | |  |  |
| 7 | 1 | |  |  | |  | |  | |  | | |  | |  | |  | | | |  | | |  | | | |  | | | |  | | | | | |  | | | | | | |  | | | |  |  |
| 7 | 1 | |  |  | |  | |  | |  | | |  | |  | |  | | | |  | | |  | | | |  | | | |  | | | | | |  | | | | | | |  | | | |  |  |
| 7 | 1 | |  |  | |  | |  | |  | | |  | |  | |  | | | |  | | |  | | | |  | | | |  | | | | | |  | | | | | | |  | | | |  |  |
| 8 | 3 | |  |  | |  | |  | |  | | |  | |  | |  | | | |  | | |  | | | |  | | | |  | | | | | |  | | | | | | |  | | | |  |  |
| 8 | 2 | |  |  | |  | |  | |  | | |  | |  | |  | | | |  | | |  | | | |  | | | |  | | | | | |  | | | | | | |  | | | |  |  |
| 8 | 2 | |  |  | |  | |  | |  | | |  | |  | |  | | | |  | | |  | | | |  | | | |  | | | | | |  | | | | | | |  | | | |  |  |
| 8 | 2 | |  |  | |  | |  | |  | | |  | |  | |  | | | |  | | |  | | | |  | | | |  | | | | | |  | | | | | | |  | | | |  |  |
| 8 | 1 | |  |  | |  | |  | |  | | |  | |  | |  | | | |  | | |  | | | |  | | | |  | | | | | |  | | | | | | |  | | | |  |  |
| 8 | 1 | |  |  | |  | |  | |  | | |  | |  | |  | | | |  | | |  | | | |  | | | |  | | | | | |  | | | | | | |  | | | |  |  |
| 8 | 1 | |  |  | |  | |  | |  | | |  | |  | |  | | | |  | | |  | | | |  | | | |  | | | | | |  | | | | | | |  | | | |  |  |
| 8 | 1 | |  |  | |  | |  | |  | | |  | |  | |  | | | |  | | |  | | | |  | | | |  | | | | | |  | | | | | | |  | | | |  |  |
| 8 | 1 | |  |  | |  | |  | |  | | |  | |  | |  | | | |  | | |  | | | |  | | | |  | | | | | |  | | | | | | |  | | | |  |  |
| 8 | 1 | |  |  | |  | |  | |  | | |  | |  | |  | | | |  | | |  | | | |  | | | |  | | | | | |  | | | | | | |  | | | |  |  |
| 8 | 1 | |  |  | |  | |  | |  | | |  | |  | |  | | | |  | | |  | | | |  | | | |  | | | | | |  | | | | | | |  | | | |  |  |
| 8 | 1 | |  |  | |  | |  | |  | | |  | |  | |  | | | |  | | |  | | | |  | | | |  | | | | | |  | | | | | | |  | | | |  |  |
| 9 | 3 | |  |  | |  | |  | |  | | |  | |  | |  | | | |  | | |  | | | |  | | | |  | | | | | |  | | | | | | |  | | | |  |  |
| 9 | 2 | |  |  | |  | |  | |  | | |  | |  | |  | | | |  | | |  | | | |  | | | |  | | | | | |  | | | | | | |  | | | |  |  |
| 9 | 1 | |  |  | |  | |  | |  | | |  | |  | |  | | | |  | | |  | | | |  | | | |  | | | | | |  | | | | | | |  | | | |  |  |
| 9 | 1 | |  |  | |  | |  | |  | | |  | |  | |  | | | |  | | |  | | | |  | | | |  | | | | | |  | | | | | | |  | | | |  |  |
| 9 | 1 | |  |  | |  | |  | |  | | |  | |  | |  | | | |  | | |  | | | |  | | | |  | | | | | |  | | | | | | |  | | | |  |  |
| 9 | 1 | |  |  | |  | |  | |  | | |  | |  | |  | | | |  | | |  | | | |  | | | |  | | | | | |  | | | | | | |  | | | |  |  |
| 9 | 1 | |  |  | |  | |  | |  | | |  | |  | |  | | | |  | | |  | | | |  | | | |  | | | | | |  | | | | | | |  | | | |  |  |
| 9 | 1 | |  |  | |  | |  | |  | | |  | |  | |  | | | |  | | |  | | | |  | | | |  | | | | | |  | | | | | | |  | | | |  |  |
| 9 | 1 | |  |  | |  | |  | |  | | |  | |  | |  | | | |  | | |  | | | |  | | | |  | | | | | |  | | | | | | |  | | | |  |  |
| 9 | 1 | |  |  | |  | |  | |  | | |  | |  | |  | | | |  | | |  | | | |  | | | |  | | | | | |  | | | | | | |  | | | |  |  |
| 10 | 3 | |  |  | |  | |  | |  | | |  | |  | |  | | | |  | | |  | | | |  | | | |  | | | | | |  | | | | | | |  | | | |  |  |
| 10 | 2 | |  |  | |  | |  | |  | | |  | |  | |  | | | |  | | |  | | | |  | | | |  | | | | | |  | | | | | | |  | | | |  |  |
| 10 | 2 | |  |  | |  | |  | |  | | |  | |  | |  | | | |  | | |  | | | |  | | | |  | | | | | |  | | | | | | |  | | | |  |  |
| 10 | 2 | |  |  | |  | |  | |  | | |  | |  | |  | | | |  | | |  | | | |  | | | |  | | | | | |  | | | | | | |  | | | |  |  |
| 10 | 1 | |  |  | |  | |  | |  | | |  | |  | |  | | | |  | | |  | | | |  | | | |  | | | | | |  | | | | | | |  | | | |  |  |
| 10 | 1 | |  |  | |  | |  | |  | | |  | |  | |  | | | |  | | |  | | | |  | | | |  | | | | | |  | | | | | | |  | | | |  |  |
| 10 | 1 | |  |  | |  | |  | |  | | |  | |  | |  | | | |  | | |  | | | |  | | | |  | | | | | |  | | | | | | |  | | | |  |  |
| 10 | 1 | |  |  | |  | |  | |  | | |  | |  | |  | | | |  | | |  | | | |  | | | |  | | | | | |  | | | | | | |  | | | |  |  |
| 10 | 1 | |  |  | |  | |  | |  | | |  | |  | |  | | | |  | | |  | | | |  | | | |  | | | | | |  | | | | | | |  | | | |  |  |
| 10 | 1 | |  |  | |  | |  | |  | | |  | |  | |  | | | |  | | |  | | | |  | | | |  | | | | | |  | | | | | | |  | | | |  |  |
| 10 | 1 | |  |  | |  | |  | |  | | |  | |  | |  | | | |  | | |  | | | |  | | | |  | | | | | |  | | | | | | |  | | | |  |  |
| 10 | 1 | |  |  | |  | |  | |  | | |  | |  | |  | | | |  | | |  | | | |  | | | |  | | | | | |  | | | | | | |  | | | |  |  |
| 11 | 4 | |  |  | |  | |  | |  | | |  | |  | |  | | | |  | | |  | | | |  | | | |  | | | | | |  | | | | | | |  | | | |  |  |
| 11 | 2 | |  |  | |  | |  | |  | | |  | |  | |  | | | |  | | |  | | | |  | | | |  | | | | | |  | | | | | | |  | | | |  |  |
| 11 | 1 | |  |  | |  | |  | |  | | |  | |  | |  | | | |  | | |  | | | |  | | | |  | | | | | |  | | | | | | |  | | | |  |  |
| 11 | 1 | |  |  | |  | |  | |  | | |  | |  | |  | | | |  | | |  | | | |  | | | |  | | | | | |  | | | | | | |  | | | |  |  |
| 12 | 5 | |  |  | |  | |  | |  | | |  | |  | |  | | | |  | | |  | | | |  | | | |  | | | | | |  | | | | | | |  | | | |  |  |
| 12 | 3 | |  |  | |  | |  | |  | | |  | |  | |  | | | |  | | |  | | | |  | | | |  | | | | | |  | | | | | | |  | | | |  |  |
| 12 | 2 | |  |  | |  | |  | |  | | |  | |  | |  | | | |  | | |  | | | |  | | | |  | | | | | |  | | | | | | |  | | | |  |  |
| 12 | 1 | |  |  | |  | |  | |  | | |  | |  | |  | | | |  | | |  | | | |  | | | |  | | | | | |  | | | | | | |  | | | |  |  |
| 12 | 1 | |  |  | |  | |  | |  | | |  | |  | |  | | | |  | | |  | | | |  | | | |  | | | | | |  | | | | | | |  | | | |  |  |
| 13 | 1 | |  |  | |  | |  | |  | | |  | |  | |  | | | |  | | |  | | | |  | | | |  | | | | | |  | | | | | | |  | | | |  |  |
| 14 | 1 | |  |  | |  | |  | |  | | |  | |  | |  | | | |  | | |  | | | |  | | | |  | | | | | |  | | | | | | |  | | | |  |  |
| 15 | 2 | |  |  | |  | |  | |  | | |  | |  | |  | | | |  | | |  | | | |  | | | |  | | | | | |  | | | | | | |  | | | |  |  |
| 15 | 1 | |  |  | |  | |  | |  | | |  | |  | |  | | | |  | | |  | | | |  | | | |  | | | | | |  | | | | | | |  | | | |  |  |
| 15 | 1 | |  |  | |  | |  | |  | | |  | |  | |  | | | |  | | |  | | | |  | | | |  | | | | | |  | | | | | | |  | | | |  |  |
| 16 | 1 | |  |  | |  | |  | |  | | |  | |  | |  | | | |  | | |  | | | |  | | | |  | | | | | |  | | | | | | |  | | | |  |  |
| 17 | 3 | |  |  | |  | |  | |  | | |  | |  | |  | | | |  | | |  | | | |  | | | |  | | | | | |  | | | | | | |  | | | |  |  |
| 18 | 1 | |  |  | |  | |  | |  | | |  | |  | |  | | | |  | | |  | | | |  | | | |  | | | | | |  | | | | | | |  | | | |  |  |
| 19 | 2 | |  |  | |  | |  | |  | | |  | |  | |  | | | |  | | |  | | | |  | | | |  | | | | | |  | | | | | | |  | | | |  |  |
| 20 | 2 | |  |  | |  | |  | |  | | |  | |  | |  | | | |  | | |  | | | |  | | | |  | | | | | |  | | | | | | |  | | | |  |  |
|  |  | |  |  | |  | |  | |  | | |  | |  | |  | | | |  | | |  | | | |  | | | |  | | | | | |  | | | | | | |  | | | |  |  |
| **Ceftriaxone users with no missed dose-days** | | |  |  | |  | |  | |  | | |  | |  | |  | | | |  | | |  | | | |  | | | |  | | | | | |  | | | | | | |  | | | |  |  |
| **Length of stay, days** | **Days missed** | | **Cases, n** |  | |  | |  | |  | | |  | |  | |  | | | |  | | |  | | | |  | | | |  | | | | | |  | | | | | | |  | | | |  |  |
| 1 | 0 | | 68 |  | |  | |  | |  | | |  | |  | |  | | | |  | | |  | | | |  | | | |  | | | | | |  | | | | | | |  | | | |  |  |
| 2 | 0 | | 56 |  | |  | |  | |  | | |  | |  | |  | | | |  | | |  | | | |  | | | |  | | | | | |  | | | | | | |  | | | |  |  |
| 3 | 0 | | 34 |  | |  | |  | |  | | |  | |  | |  | | | |  | | |  | | | |  | | | |  | | | | | |  | | | | | | |  | | | |  |  |
| 4 | 0 | | 26 |  | |  | |  | |  | | |  | |  | |  | | | |  | | |  | | | |  | | | |  | | | | | |  | | | | | | |  | | | |  |  |
| 5 | 0 | | 27 |  | |  | |  | |  | | |  | |  | |  | | | |  | | |  | | | |  | | | |  | | | | | |  | | | | | | |  | | | |  |  |
| 6 | 0 | | 7 |  | |  | |  | |  | | |  | |  | |  | | | |  | | |  | | | |  | | | |  | | | | | |  | | | | | | |  | | | |  |  |
| 7 | 0 | | 7 |  | |  | |  | |  | | |  | |  | |  | | | |  | | |  | | | |  | | | |  | | | | | |  | | | | | | |  | | | |  |  |
| 8 | 0 | | 2 |  | |  | |  | |  | | |  | |  | |  | | | |  | | |  | | | |  | | | |  | | | | | |  | | | | | | |  | | | |  |  |
|  |  | |  |  | |  | |  | |  | | |  | |  | |  | | | |  | | |  | | | |  | | | |  | | | | | |  | | | | | | |  | | | |  |  |
| **Missed dose-day, n** | | | 143 | 35 | | 22 | | 10 | | 4 | | | 6 | | 2 | | 3 | | | | 1 | | | 0 | | | |  | | | |  | | | | | |  | | | | | | |  | | | |  |  |
| **Dose-day data unavailable, n1** | | | 0 | 68 | | 154 | | 220 | | 278 | | | 334 | | 362 | | 382 | | | | 391 | | | 393 | | | |  | | | |  | | | | | |  | | | | | | |  | | | |  |  |
| **Dose-day data available, N*** | | | 398 | 330 | | 244 | | 178 | | 120 | | | 64 | | 36 | | 16 | | | | 7 | | | 5 | | | |  | | | |  | | | | | |  | | | | | | |  | | | |  |  |
| **Missed dose-day, (n/N)%** | | | 36 | 11 | | 9 | | 6 | | 3 | | | 9 | | 6 | | 19 | | | | 14 | | | 0 | | | |  | | | |  | | | | | |  | | | | | | |  | | | |  |  |
| ***N = (398-n1) or (The 398 ceftriaxone users) - (Number of patients who didn’t have ceftriaxone-dose-day data)** | | | | | | | | | | | | | | | | | | | | | | | | | | | |  | | | |  | | | | | |  | | | | | | |  | | | |  |  |
|  | | | | |  | | | |  | | | |  |  | |  | |  | | |  |  | | |  | | | |  | |  | | | |  | |  | |  | | | | | |  | | | |  |  |
| **Missed dose-days of intravenous or oral metronidazole among 246 hospitalized patients who received in-hospital metronidazole, Uganda, 2014** | | | | | | | | | | | | | | | | | | | | | | | | | | | | | | | | |  | | | | | | | | | | | | |  | |  | | |
| **Length of stay, days** | **Days missed** | | **Day 1** | **Day 2** | | **Day 3** | **Day 4** | | | | **Day 5** | **Day 6** | | | **Day 7** | | **Day 8** | | | **Day 9** | | | **Day 10** | | | | | **Day 11** | | | | |  | | | | | | | | | | | | |  | |  | | |
| 2 | 1 | |  |  | |  |  | | | |  |  | | |  | |  | | |  | | |  | | | | |  | | | | |  | | | | | | | | | | | | |  | |  | | |
| 2 | 1 | |  |  | |  |  | | | |  |  | | |  | |  | | |  | | |  | | | | |  | | | | |  | | | | | | | | | | | | |  | |  | | |
| 2 | 1 | |  |  | |  |  | | | |  |  | | |  | |  | | |  | | |  | | | | |  | | | | |  | | | | | | | | | | | | |  | |  | | |
| 2 | 1 | |  |  | |  |  | | | |  |  | | |  | |  | | |  | | |  | | | | |  | | | | |  | | | | | | | | | | | | | **KEY** | |  | | |
| 2 | 1 | |  |  | |  |  | | | |  |  | | |  | |  | | |  | | |  | | | | |  | | | | |  | | | | | | | | | | | | |  | | All doses missed | | |
| 2 | 1 | |  |  | |  |  | | | |  |  | | |  | |  | | |  | | |  | | | | |  | | | | |  | | | | | | | | | | | | |  | |  | | |
| 2 | 1 | |  |  | |  |  | | | |  |  | | |  | |  | | |  | | |  | | | | |  | | | | |  | | | | | | | | | | | | |  | |  | | |
| 3 | 1 | |  |  | |  |  | | | |  |  | | |  | |  | | |  | | |  | | | | |  | | | | |  | | | | | | | | | | | | |  | |  | | |
| 3 | 1 | |  |  | |  |  | | | |  |  | | |  | |  | | |  | | |  | | | | |  | | | | |  | | | | | | | | | | | | |  | |  | | |
| 3 | 1 | |  |  | |  |  | | | |  |  | | |  | |  | | |  | | |  | | | | |  | | | | |  | | | | | | | | | | | | |  | |  | | |
| 3 | 1 | |  |  | |  |  | | | |  |  | | |  | |  | | |  | | |  | | | | |  | | | | |  | | | | | | | | | | | | |  | |  | | |
| 3 | 1 | |  |  | |  |  | | | |  |  | | |  | |  | | |  | | |  | | | | |  | | | | |  | | | | | | | | | | | | |  | |  | | |
| 3 | 1 | |  |  | |  |  | | | |  |  | | |  | |  | | |  | | |  | | | | |  | | | | |  | | | | | | | | | | | | |  | |  | | |
| 3 | 1 | |  |  | |  |  | | | |  |  | | |  | |  | | |  | | |  | | | | |  | | | | |  | | | | | | | | | | | | |  | |  | | |
| 3 | 1 | |  |  | |  |  | | | |  |  | | |  | |  | | |  | | |  | | | | |  | | | | |  | | | | | | | | | | | | |  | |  | | |
| 3 | 1 | |  |  | |  |  | | | |  |  | | |  | |  | | |  | | |  | | | | |  | | | | |  | | | | | | | | | | | | |  | |  | | |
| 3 | 1 | |  |  | |  |  | | | |  |  | | |  | |  | | |  | | |  | | | | |  | | | | |  | | | | | | | | | | | | |  | |  | | |
| 4 | 2 | |  |  | |  |  | | | |  |  | | |  | |  | | |  | | |  | | | | |  | | | | |  | | | | | | | | | | | | |  | |  | | |
| 4 | 2 | |  |  | |  |  | | | |  |  | | |  | |  | | |  | | |  | | | | |  | | | | |  | | | | | | | | | | | | |  | |  | | |
| 4 | 1 | |  |  | |  |  | | | |  |  | | |  | |  | | |  | | |  | | | | |  | | | | |  | | | | | | | | | | | | |  | |  | | |
| 4 | 1 | |  |  | |  |  | | | |  |  | | |  | |  | | |  | | |  | | | | |  | | | | |  | | | | | | | | | | | | |  | |  | | |
| 4 | 1 | |  |  | |  |  | | | |  |  | | |  | |  | | |  | | |  | | | | |  | | | | |  | | | | | | | | | | | | |  | |  | | |
| 4 | 1 | |  |  | |  |  | | | |  |  | | |  | |  | | |  | | |  | | | | |  | | | | |  | | | | | | | | | | | | |  | |  | | |
| 4 | 1 | |  |  | |  |  | | | |  |  | | |  | |  | | |  | | |  | | | | |  | | | | |  | | | | | | | | | | | | |  | |  | | |
| 4 | 1 | |  |  | |  |  | | | |  |  | | |  | |  | | |  | | |  | | | | |  | | | | |  | | | | | | | | | | | | |  | |  | | |
| 4 | 1 | |  |  | |  |  | | | |  |  | | |  | |  | | |  | | |  | | | | |  | | | | |  | | | | | | | | | | | | |  | |  | | |
| 4 | 1 | |  |  | |  |  | | | |  |  | | |  | |  | | |  | | |  | | | | |  | | | | |  | | | | | | | | | | | | |  | |  | | |
| 4 | 1 | |  |  | |  |  | | | |  |  | | |  | |  | | |  | | |  | | | | |  | | | | |  | | | | | | | | | | | | |  | |  | | |
| 4 | 1 | |  |  | |  |  | | | |  |  | | |  | |  | | |  | | |  | | | | |  | | | | |  | | | | | | | | | | | | |  | |  | | |
| 4 | 1 | |  |  | |  |  | | | |  |  | | |  | |  | | |  | | |  | | | | |  | | | | |  | | | | | | | | | | | | |  | |  | | |
| 4 | 1 | |  |  | |  |  | | | |  |  | | |  | |  | | |  | | |  | | | | |  | | | | |  | | | | | | | | | | | | |  | |  | | |
| 5 | 2 | |  |  | |  |  | | | |  |  | | |  | |  | | |  | | |  | | | | |  | | | | |  | | | | | | | | | | | | |  | |  | | |
| 5 | 2 | |  |  | |  |  | | | |  |  | | |  | |  | | |  | | |  | | | | |  | | | | |  | | | | | | | | | | | | |  | |  | | |
| 5 | 1 | |  |  | |  |  | | | |  |  | | |  | |  | | |  | | |  | | | | |  | | | | |  | | | | | | | | | | | | |  | |  | | |
| 5 | 1 | |  |  | |  |  | | | |  |  | | |  | |  | | |  | | |  | | | | |  | | | | |  | | | | | | | | | | | | |  | |  | | |
| 5 | 1 | |  |  | |  |  | | | |  |  | | |  | |  | | |  | | |  | | | | |  | | | | |  | | | | | | | | | | | | |  | |  | | |
| 5 | 1 | |  |  | |  |  | | | |  |  | | |  | |  | | |  | | |  | | | | |  | | | | |  | | | | | | | | | | | | |  | |  | | |
| 5 | 1 | |  |  | |  |  | | | |  |  | | |  | |  | | |  | | |  | | | | |  | | | | |  | | | | | | | | | | | | |  | |  | | |
| 5 | 1 | |  |  | |  |  | | | |  |  | | |  | |  | | |  | | |  | | | | |  | | | | |  | | | | | | | | | | | | |  | |  | | |
| 5 | 1 | |  |  | |  |  | | | |  |  | | |  | |  | | |  | | |  | | | | |  | | | | |  | | | | | | | | | | | | |  | |  | | |
| 5 | 1 | |  |  | |  |  | | | |  |  | | |  | |  | | |  | | |  | | | | |  | | | | |  | | | | | | | | | | | | |  | |  | | |
| 5 | 1 | |  |  | |  |  | | | |  |  | | |  | |  | | |  | | |  | | | | |  | | | | |  | | | | | | | | | | | | |  | |  | | |
| 6 | 3 | |  |  | |  |  | | | |  |  | | |  | |  | | |  | | |  | | | | |  | | | | |  | | | | | | | | | | | | |  | |  | | |
| 6 | 3 | |  |  | |  |  | | | |  |  | | |  | |  | | |  | | |  | | | | |  | | | | |  | | | | | | | | | | | | |  | |  | | |
| 6 | 1 | |  |  | |  |  | | | |  |  | | |  | |  | | |  | | |  | | | | |  | | | | |  | | | | | | | | | | | | |  | |  | | |
| 6 | 1 | |  |  | |  |  | | | |  |  | | |  | |  | | |  | | |  | | | | |  | | | | |  | | | | | | | | | | | | |  | |  | | |
| 6 | 1 | |  |  | |  |  | | | |  |  | | |  | |  | | |  | | |  | | | | |  | | | | |  | | | | | | | | | | | | |  | |  | | |
| 6 | 1 | |  |  | |  |  | | | |  |  | | |  | |  | | |  | | |  | | | | |  | | | | |  | | | | | | | | | | | | |  | |  | | |
| 6 | 1 | |  |  | |  |  | | | |  |  | | |  | |  | | |  | | |  | | | | |  | | | | |  | | | | | | | | | | | | |  | |  | | |
| 6 | 1 | |  |  | |  |  | | | |  |  | | |  | |  | | |  | | |  | | | | |  | | | | |  | | | | | | | | | | | | |  | |  | | |
| 7 | 2 | |  |  | |  |  | | | |  |  | | |  | |  | | |  | | |  | | | | |  | | | | |  | | | | | | | | | | | | |  | |  | | |
| 7 | 2 | |  |  | |  |  | | | |  |  | | |  | |  | | |  | | |  | | | | |  | | | | |  | | | | | | | | | | | | |  | |  | | |
| 7 | 2 | |  |  | |  |  | | | |  |  | | |  | |  | | |  | | |  | | | | |  | | | | |  | | | | | | | | | | | | |  | |  | | |
| 7 | 1 | |  |  | |  |  | | | |  |  | | |  | |  | | |  | | |  | | | | |  | | | | |  | | | | | | | | | | | | |  | |  | | |
| 7 | 1 | |  |  | |  |  | | | |  |  | | |  | |  | | |  | | |  | | | | |  | | | | |  | | | | | | | | | | | | |  | |  | | |
| 7 | 1 | |  |  | |  |  | | | |  |  | | |  | |  | | |  | | |  | | | | |  | | | | |  | | | | | | | | | | | | |  | |  | | |
| 7 | 1 | |  |  | |  |  | | | |  |  | | |  | |  | | |  | | |  | | | | |  | | | | |  | | | | | | | | | | | | |  | |  | | |
| 7 | 1 | |  |  | |  |  | | | |  |  | | |  | |  | | |  | | |  | | | | |  | | | | |  | | | | | | | | | | | | |  | |  | | |
| 7 | 1 | |  |  | |  |  | | | |  |  | | |  | |  | | |  | | |  | | | | |  | | | | |  | | | | | | | | | | | | |  | |  | | |
| 8 | 2 | |  |  | |  |  | | | |  |  | | |  | |  | | |  | | |  | | | | |  | | | | |  | | | | | | | | | | | | |  | |  | | |
| 8 | 2 | |  |  | |  |  | | | |  |  | | |  | |  | | |  | | |  | | | | |  | | | | |  | | | | | | | | | | | | |  | |  | | |
| 8 | 2 | |  |  | |  |  | | | |  |  | | |  | |  | | |  | | |  | | | | |  | | | | |  | | | | | | | | | | | | |  | |  | | |
| 8 | 1 | |  |  | |  |  | | | |  |  | | |  | |  | | |  | | |  | | | | |  | | | | |  | | | | | | | | | | | | |  | |  | | |
| 8 | 1 | |  |  | |  |  | | | |  |  | | |  | |  | | |  | | |  | | | | |  | | | | |  | | | | | | | | | | | | |  | |  | | |
| 8 | 1 | |  |  | |  |  | | | |  |  | | |  | |  | | |  | | |  | | | | |  | | | | |  | | | | | | | | | | | | |  | |  | | |
| 9 | 3 | |  |  | |  |  | | | |  |  | | |  | |  | | |  | | |  | | | | |  | | | | |  | | | | | | | | | | | | |  | |  | | |
| 9 | 2 | |  |  | |  |  | | | |  |  | | |  | |  | | |  | | |  | | | | |  | | | | |  | | | | | | | | | | | | |  | |  | | |
| 9 | 1 | |  |  | |  |  | | | |  |  | | |  | |  | | |  | | |  | | | | |  | | | | |  | | | | | | | | | | | | |  | |  | | |
| 10 | 4 | |  |  | |  |  | | | |  |  | | |  | |  | | |  | | |  | | | | |  | | | | |  | | | | | | | | | | | | |  | |  | | |
| 10 | 1 | |  |  | |  |  | | | |  |  | | |  | |  | | |  | | |  | | | | |  | | | | |  | | | | | | | | | | | | |  | |  | | |
| 11 | 1 | |  |  | |  |  | | | |  |  | | |  | |  | | |  | | |  | | | | |  | | | | |  | | | | | | | | | | | | |  | |  | | |
| 11 | 1 | |  |  | |  |  | | | |  |  | | |  | |  | | |  | | |  | | | | |  | | | | |  | | | | | | | | | | | | |  | |  | | |
| 12 | 3 | |  |  | |  |  | | | |  |  | | |  | |  | | |  | | |  | | | | |  | | | | |  | | | | | | | | | | | | |  | |  | | |
| 14 | 2 | |  |  | |  |  | | | |  |  | | |  | |  | | |  | | |  | | | | |  | | | | |  | | | | | | | | | | | | |  | |  | | |
| 14 | 1 | |  |  | |  |  | | | |  |  | | |  | |  | | |  | | |  | | | | |  | | | | |  | | | | | | | | | | | | |  | |  | | |
| 15 | 1 | |  |  | |  |  | | | |  |  | | |  | |  | | |  | | |  | | | | |  | | | | |  | | | | | | | | | | | | |  | |  | | |
| 20 | 1 | |  |  | |  |  | | | |  |  | | |  | |  | | |  | | |  | | | | |  | | | | |  | | | | | | | | | | | | |  | |  | | |
|  |  | |  |  | |  |  | | | |  |  | | |  | |  | | |  | | |  | | | | |  | | | | |  | | | | | | | | | | | | |  | |  | | |
| **Metronidazole users with no missed dose-days** | | |  |  | |  |  | | | |  |  | | |  | |  | | |  | | |  | | | | |  | | | | |  | | | | | | | | | | | | |  | |  | | |
| **Length of stay, days** | **Days missed** | | **Cases, n** |  | |  |  | | | |  |  | | |  | |  | | |  | | |  | | | | |  | | | | |  | | | | | | | | | | | | |  | |  | | |
| 1 | 0 | | 50 |  | |  |  | | | |  |  | | |  | |  | | |  | | |  | | | | |  | | | | |  | | | | | | | | | | | | |  | |  | | |
| 2 | 0 | | 46 |  | |  |  | | | |  |  | | |  | |  | | |  | | |  | | | | |  | | | | |  | | | | | | | | | | | | |  | |  | | |
| 3 | 0 | | 28 |  | |  |  | | | |  |  | | |  | |  | | |  | | |  | | | | |  | | | | |  | | | | | | | | | | | | |  | |  | | |
| 4 | 0 | | 26 |  | |  |  | | | |  |  | | |  | |  | | |  | | |  | | | | |  | | | | |  | | | | | | | | | | | | |  | |  | | |
| 5 | 0 | | 6 |  | |  |  | | | |  |  | | |  | |  | | |  | | |  | | | | |  | | | | |  | | | | | | | | | | | | |  | |  | | |
| 6 | 0 | | 4 |  | |  |  | | | |  |  | | |  | |  | | |  | | |  | | | | |  | | | | |  | | | | | | | | | | | | |  | |  | | |
| 7 | 0 | | 3 |  | |  |  | | | |  |  | | |  | |  | | |  | | |  | | | | |  | | | | |  | | | | | | | | | | | | |  | |  | | |
| 8 | 0 | | 4 |  | |  |  | | | |  |  | | |  | |  | | |  | | |  | | | | |  | | | | |  | | | | | | | | | | | | |  | |  | | |
| 9 | 0 | | 1 |  | |  |  | | | |  |  | | |  | |  | | |  | | |  | | | | |  | | | | |  | | | | | | | | | | | | |  | |  | | |
| 7 | --- | | 1 |  | |  |  | | | |  |  | | |  | |  | | |  | | |  | | | | |  | | | | |  | | | | | | | | | | | | |  | |  | | |
|  |  | |  |  | |  |  | | | |  |  | | |  | |  | | |  | | |  | | | | |  | | | | |  | | | | | | | | | | | | |  | |  | | |
| **Missed dose-day, n** | | | 67 | 18 | | 7 | 1 | | | | 4 | 3 | | | 0 | | 0 | | | 0 | | | 0 | | | | | 0 | | | | |  | | | | | | | | | | | | |  | |  | | |
| **Dose-day data unavailable, n1^╪^** | | | 1 | 52 | | 114 | 158 | | | | 196 | 215 | | | 229 | | 235 | | | 242 | | | 244 | | | | | 245 | | | | |  | | | | | | | | | |  |  | | | | | |  |
| **Dose-day data available, N*** | | | 245 | 194 | | 132 | 88 | | | | 50 | 31 | | | 17 | | 11 | | | 4 | | | 2 | | | | | 1 | | | | |  | | | | | | | | | |  |  | | | | | |  |
| **Missed dose-day, (n/N)%** | | | 27 | 9 | | 5 | 1 | | | | 8 | 10 | | | 0 | | 0 | | | 0 | | | 0 | | | | | 0 | | | | |  | | | | | | | | | |  |  | | | | | |  |
| **^╪^One patient received metronidazole but no drug administration details were available for the patient** | | | | | | | | | | | | | | | | | | | | | | | | | | | | | | | | |  | | | | | | | | | | | | |  | |  | | |
| ***N = (245-n1) or (The 245 metronidazole users) - (Patients who didn’t have metronidazole-dose-day data)** | | | | | | | | | | | | | | | | | | | | | | | | | | | | | | | | |  | | | | | | | | | | | | |  | |  | | |
|  | | | | | | | | | | | | | | | | | | | | | | | | | | | | | | | | |  | | | | | | | | | | | | |  | |  | | |
| **Missed dose-days of intravenous or oral ciprofloxacin among 114 hospitalized patients who received in-hospital ciprofloxacin, Uganda, 2014** | | | | | | | | | | | | | | | | | | | | | | | | | | | |  | | | | | | | | | | | |  |  | | | | | |  |  |  |  |
| **Length of stay, days** | **Days missed** | | **Day 1** | **Day 2** | | **Day 3** | **Day 4** | | | | **Day 5** | **Day 6** | | | **Day 7** | | **Day 8** | | | **Day 9** | | | **Day 10** | | | | |  | | | | | | | | | | | |  |  | | | | | |  |  |  |  |
| 2 | 1 | |  |  | |  |  | | | |  |  | | |  | |  | | |  | | |  | | | | |  | | | | | | | | | | | |  |  | | | | | |  |  |  |  |
| 2 | 1 | |  |  | |  |  | | | |  |  | | |  | |  | | |  | | |  | | | | |  | | | | | | | | | | | |  |  | | | | | |  |  |  |  |
| 3 | 1 | |  |  | |  |  | | | |  |  | | |  | |  | | |  | | |  | | | | |  | | | | | | | | | | | |  |  | | | | | |  |  |  |  |
| 3 | 1 | |  |  | |  |  | | | |  |  | | |  | |  | | |  | | |  | | | | |  | | | | | | | | | | | |  |  | | | | | |  |  |  |  |
| 3 | 1 | |  |  | |  |  | | | |  |  | | |  | |  | | |  | | |  | | | | |  |  |  |  |  |  |  |  |  |  |  |  |  |  |  |  |  |  |  |  |  |  |  |
| 4 | 2 | |  |  | |  |  | | | |  |  | | |  | |  | | |  | | |  | | | | |  |  |  |  |  |  |  |  |  |  |  |  |  |  |  |  |  |  |  |  |  |  |  |
| 4 | 2 | |  |  | |  |  | | | |  |  | | |  | |  | | |  | | |  | | | | |  |  |  |  |  |  |  |  |  |  |  |  |  |  |  |  |  |  |  |  |  |  |  |
| 4 | 1 | |  |  | |  |  | | | |  |  | | |  | |  | | |  | | |  | | | | |  |  |  |  |  |  |  |  |  |  |  |  |  |  |  |  |  |  |  |  |  |  |  |
| 4 | 1 | |  |  | |  |  | | | |  |  | | |  | |  | | |  | | |  | | | | |  |  |  |  |  |  |  |  |  |  |  |  |  |  |  |  |  |  |  |  |  |  |  |
| 4 | 1 | |  |  | |  |  | | | |  |  | | |  | |  | | |  | | |  | | | | |  | | | | | | | | | | | |  |  | | | | | |  |  |  |  |
| 4 | 1 | |  |  | |  |  | | | |  |  | | |  | |  | | |  | | |  | | | | |  | | | | | | | | | | | |  |  | | | | | |  |  |  |  |
| 4 | 1 | |  |  | |  |  | | | |  |  | | |  | |  | | |  | | |  | | | | |  | | | | | | | | | | | |  |  | | | | | |  |  |  |  |
| 5 | 3 | |  |  | |  |  | | | |  |  | | |  | |  | | |  | | |  | | | | |  | | | | | | | | | | | |  |  | | | | | |  |  |  |  |
| 5 | 2 | |  |  | |  |  | | | |  |  | | |  | |  | | |  | | |  | | | | |  | | | | | | | | | | | |  |  | | | | | |  |  |  |  |
| 5 | 2 | |  |  | |  |  | | | |  |  | | |  | |  | | |  | | |  | | | | |  | | | | | | | | | | | |  |  | | | | | |  |  |  |  |
| 5 | 1 | |  |  | |  |  | | | |  |  | | |  | |  | | |  | | |  | | | | |  | | | | | | | | | | | |  |  | | | | | |  |  |  |  |
| 5 | 1 | |  |  | |  |  | | | |  |  | | |  | |  | | |  | | |  | | | | |  | | | | | | | | | | | |  |  | | | | | |  |  |  |  |
| 5 | 1 | |  |  | |  |  | | | |  |  | | |  | |  | | |  | | |  | | | | |  | | | | | | | | | | | |  |  | | | | | |  |  |  |  |
| 6 | 2 | |  |  | |  |  | | | |  |  | | |  | |  | | |  | | |  | | | | |  | | | | | | | | | | | |  |  | | | | | |  |  |  |  |
| 6 | 1 | |  |  | |  |  | | | |  |  | | |  | |  | | |  | | |  | | | | |  | | | | | | | | | | | |  |  | | | | | |  |  |  |  |
| 6 | 1 | |  |  | |  |  | | | |  |  | | |  | |  | | |  | | |  | | | | |  | | | | | | | | | | | |  |  | | | | | |  |  |  |  |
| 6 | 1 | |  |  | |  |  | | | |  |  | | |  | |  | | |  | | |  | | | | |  | | | | | | | | | | | |  |  | | | | | |  |  |  |  |
| 6 | 1 | |  |  | |  |  | | | |  |  | | |  | |  | | |  | | |  | | | | |  | | | | | | | | | | | |  |  | | | | | |  |  |  |  |
| 6 | 1 | |  |  | |  |  | | | |  |  | | |  | |  | | |  | | |  | | | | |  | | | | | | | | | | | |  |  | | | | | |  |  |  |  |
| 6 | 1 | |  |  | |  |  | | | |  |  | | |  | |  | | |  | | |  | | | | |  | | | | | | | | | | | |  |  | | | | | |  |  |  |  |
| 6 | 1 | |  |  | |  |  | | | |  |  | | |  | |  | | |  | | |  | | | | |  | | | | | | | | | | | |  |  | | | | | |  |  |  |  |
| 7 | 2 | |  |  | |  |  | | | |  |  | | |  | |  | | |  | | |  | | | | |  | | | | | | | | | | | |  |  | | | | | |  |  |  |  |
| 7 | 2 | |  |  | |  |  | | | |  |  | | |  | |  | | |  | | |  | | | | |  | | | | | | | | | | | |  |  | | | | | |  |  |  |  |
| 7 | 1 | |  |  | |  |  | | | |  |  | | |  | |  | | |  | | |  | | | | |  | | | | | | | | | | | |  |  | | | | | |  |  |  |  |
| 7 | 1 | |  |  | |  |  | | | |  |  | | |  | |  | | |  | | |  | | | | |  | | | | | | | | | | | |  |  | | | | | |  |  |  |  |
| 8 | 2 | |  |  | |  |  | | | |  |  | | |  | |  | | |  | | |  | | | | |  | | | | | | | | | | | |  |  | | | | | |  |  |  |  |
| 8 | 1 | |  |  | |  |  | | | |  |  | | |  | |  | | |  | | |  | | | | |  | | | | | | | | | | | |  |  | | | | | |  |  |  |  |
| 9 | 3 | |  |  | |  |  | | | |  |  | | |  | |  | | |  | | |  | | | | |  | | | | | | | | | | | |  |  | | | | | |  |  |  |  |
| 9 | 3 | |  |  | |  |  | | | |  |  | | |  | |  | | |  | | |  | | | | |  | | | | | | | | | | | |  |  | | | | | |  |  |  |  |
| 10 | 3 | |  |  | |  |  | | | |  |  | | |  | |  | | |  | | |  | | | | |  | | | | | | | | | | | |  |  | | | | | |  |  |  |  |
| 10 | 1 | |  |  | |  |  | | | |  |  | | |  | |  | | |  | | |  | | | | |  | | | | | | | | | | | |  |  | | | | | |  |  |  |  |
| 10 | 1 | |  |  | |  |  | | | |  |  | | |  | |  | | |  | | |  | | | | |  | | | | | | | | | | | |  |  | | | | | |  |  |  |  |
| 12 | 1 | |  |  | |  |  | | | |  |  | | |  | |  | | |  | | |  | | | | |  | | | | | | | | | | | |  |  | | | | | |  |  |  |  |
| 14 | 3 | |  |  | |  |  | | | |  |  | | |  | |  | | |  | | |  | | | | |  | | | | | | | | | | | |  |  | | | | | |  |  |  |  |
| 14 | 1 | |  |  | |  |  | | | |  |  | | |  | |  | | |  | | |  | | | | |  | | | | | | | | | | | |  |  | | | | | |  |  |  |  |
| 15 | 1 | |  |  | |  |  | | | |  |  | | |  | |  | | |  | | |  | | | | |  | | | | | | | | | | | |  |  | | | | | |  |  |  |  |
| 15 | 1 | |  |  | |  |  | | | |  |  | | |  | |  | | |  | | |  | | | | |  | | | | | | | | | | | |  |  | | | | | |  |  |  |  |
| 18 | 1 | |  |  | |  |  | | | |  |  | | |  | |  | | |  | | |  | | | | |  | | | | | | | | | | | |  |  | | | | | |  |  |  |  |
|  |  | |  |  | |  |  | | | |  |  | | |  | |  | | |  | | |  | | | | |  | | | | | | | | | | | |  |  | | | | | |  |  |  |  |
| **Ciprofloxacin users with no missed dose-days** | | |  |  | |  |  | | | |  |  | | |  | |  | | |  | | |  | | | | |  | | | | | | | | | | | |  |  | | | | | |  |  |  |  |
| **Length of stay, days** | **Days missed** | | **Cases, n** |  | |  |  | | | |  |  | | |  | |  | | |  | | |  | | | | |  | | | | | | | | | | | |  |  | | | | | |  |  |  |  |
| 1 | 0 | | 20 |  | |  |  | | | |  |  | | |  | |  | | |  | | |  | | | | |  | | | | | | | | | | | |  |  | | | | | |  |  |  |  |
| 2 | 0 | | 29 |  | |  |  | | | |  |  | | |  | |  | | |  | | |  | | | | |  | | | | | | | | | | | |  |  | | | | | |  |  |  |  |
| 3 | 0 | | 10 |  | |  |  | | | |  |  | | |  | |  | | |  | | |  | | | | |  | | | | | | | | | | | |  |  | | | | | |  |  |  |  |
| 4 | 0 | | 8 |  | |  |  | | | |  |  | | |  | |  | | |  | | |  | | | | |  | | | | | | | | | | | |  |  | | | | | |  |  |  |  |
| 5 | 0 | | 2 |  | |  |  | | | |  |  | | |  | |  | | |  | | |  | | | | |  | | | | | | | | | | | |  |  | | | | | |  |  |  |  |
| 6 | 0 | | 1 |  | |  |  | | | |  |  | | |  | |  | | |  | | |  | | | | |  | | | | | | | | | | | |  |  | | | | | |  |  |  |  |
| 8 | 0 | | 1 |  | |  |  | | | |  |  | | |  | |  | | |  | | |  | | | | |  | | | | | | | | | | | |  |  | | | | | |  |  |  |  |
|  |  | |  |  | |  |  | | | |  |  | | |  | |  | | |  | | |  | | | | |  | | | | | | | | | | | |  |  | | | | | |  |  |  |  |
| **Missed dose-day, n** | | | 40 | 9 | | 5 | 3 | | | | 2 | 2 | | | 0 | | 0 | | | 0 | | | 0 | | | | |  | | | | | | | | | | | |  |  | | | | | |  |  |  |  |
| **Dose-day data unavailable, n1** | | | 0 | 20 | | 56 | 79 | | | | 94 | 103 | | | 107 | | 110 | | | 111 | | | 112 | | | | |  | | | | | | | | | | | |  |  | | | | | |  |  |  |  |
| **Dose-day data available, N*** | | | 114 | 94 | | 58 | 35 | | | | 20 | 11 | | | 7 | | 4 | | | 3 | | | 2 | | | | |  | | | | | | | | | | | |  |  | | | | | |  |  |  |  |
| **Missed dose-day, (n/N)%** | | | 35 | 10 | | 9 | 9 | | | | 10 | 18 | | | 0 | | 0 | | | 0 | | | 0 | | | | |  | | | | | | | | | | | |  |  | | | | | |  |  |  |  |
| ***N = (114-n1) or (The 114 ciprofloxacin users) - (Patients who didn’t have ciprofloxacin-dose-day data)** | | | | | | | | | | | | | | | | | | | | | | | | | | | |  | | | | | | | | | | | |  |  | | | | | |  |  |  |  |
|  | | | | | | | | | | | | | | | | | | | | | | | | | | | |  | | | | | | | | | | | |  |  | | | | | |  |  |  |  |
| **Missed dose-days of oral amoxicillin among 57 hospitalized patients who received in-hospital amoxicillin, Uganda, 2014** | | | | | | | | | | | | | | | | | | | | | | | | | | | |  | | | | | | | | | | | | | |  |  |  |  |  |  |  |  |  |
| **Length of stay, days** | **Days missed** | | **Day 1** | **Day 2** | | **Day 3** | **Day 4** | | | | **Day 5** | **Day 6** | | | **Day 7** | |  | | |  | | |  | | | | |  |  |  |  |  |  |  |  |  |  |  |  |  |  |  |  |  |  |  |  |  |  |  |
| 3 | 1 | |  |  | |  |  | | | |  |  | | |  | |  | | |  | | |  | | | | |  |  |  |  |  |  |  |  |  |  |  |  |  |  |  |  |  |  |  |  |  |  |  |
| 3 | 1 | |  |  | |  |  | | | |  |  | | |  | |  | | |  | | |  | | | | |  |  |  |  |  |  |  |  |  |  |  |  |  |  |  |  |  |  |  |  |  |  |  |
| 3 | 1 | |  |  | |  |  | | | |  |  | | |  | |  | | |  | | |  | | | | |  |  |  |  |  |  |  |  |  |  |  |  |  |  |  |  |  |  |  |  |  |  |  |
| 4 | 1 | |  |  | |  |  | | | |  |  | | |  | |  | | |  | | |  | | | | |  |  |  |  |  |  |  |  |  |  |  |  |  |  |  |  |  |  |  |  |  |  |  |
| 4 | 1 | |  |  | |  |  | | | |  |  | | |  | |  | | |  | | |  | | | | |  |  |  |  |  |  |  |  |  |  |  |  |  |  |  |  |  |  |  |  |  |  |  |
| 6 | 1 | |  |  | |  |  | | | |  |  | | |  | |  | | |  | | |  | | | | |  |  |  |  |  |  |  |  |  |  |  |  |  |  |  |  |  |  |  |  |  |  |  |
|  |  | |  |  | |  |  | | | |  |  | | |  | |  | | |  | | |  | | | | |  |  |  |  |  |  |  |  |  |  |  |  |  |  |  |  |  |  |  |  |  |  |  |
| **Amoxicillin users with no missed dose-days** | | |  |  | |  |  | | | |  |  | | |  | |  | | |  | | |  | | | | |  |  |  |  |  |  |  |  |  |  |  |  |  |  |  |  |  |  |  |  |  |  |  |
| **Length of stay, days** | **Days missed** | | **Cases, n** |  | |  |  | | | |  |  | | |  | |  | | |  | | |  | | | | |  |  |  |  |  |  |  |  |  |  |  |  |  |  |  |  |  |  |  |  |  |  |  |
| 1 | 0 | | 11 |  | |  |  | | | |  |  | | |  | |  | | |  | | |  | | | | |  |  |  |  |  |  |  |  |  |  |  |  |  |  |  |  |  |  |  |  |  |  |  |
| 2 | 0 | | 17 |  | |  |  | | | |  |  | | |  | |  | | |  | | |  | | | | |  |  |  |  |  |  |  |  |  |  |  |  |  |  |  |  |  |  |  |  |  |  |  |
| 3 | 0 | | 8 |  | |  |  | | | |  |  | | |  | |  | | |  | | |  | | | | |  |  |  |  |  |  |  |  |  |  |  |  |  |  |  |  |  |  |  |  |  |  |  |
| 4 | 0 | | 8 |  | |  |  | | | |  |  | | |  | |  | | |  | | |  | | | | |  |  |  |  |  |  |  |  |  |  |  |  |  |  |  |  |  |  |  |  |  |  |  |
| 5 | 0 | | 4 |  | |  |  | | | |  |  | | |  | |  | | |  | | |  | | | | |  |  |  |  |  |  |  |  |  |  |  |  |  |  |  |  |  |  |  |  |  |  |  |
| 6 | 0 | | 1 |  | |  |  | | | |  |  | | |  | |  | | |  | | |  | | | | |  |  |  |  |  |  |  |  |  |  |  |  |  |  |  |  |  |  |  |  |  |  |  |
| 7 | 0 | | 1 |  | |  |  | | | |  |  | | |  | |  | | |  | | |  | | | | |  |  |  |  |  |  |  |  |  |  |  |  |  |  |  |  |  |  |  |  |  |  |  |
|  |  | |  |  | |  |  | | | |  |  | | |  | |  | | |  | | |  | | | | |  |  |  |  |  |  |  |  |  |  |  |  |  |  |  |  |  |  |  |  |  |  |  |
| **Missed dose-day, n** | | | 6 | 0 | | 0 | 0 | | | | 0 | 0 | | | 0 | |  | | |  | | |  | | | | |  |  |  |  |  |  |  |  |  |  |  |  |  |  |  |  |  |  |  |  |  |  |  |
| **Dose-day data unavailable, n1** | | | 0 | 12 | | 36 | 42 | | | | 51 | 55 | | | 56 | |  | | |  | | |  | | | | |  |  |  |  |  |  |  |  |  |  |  |  |  |  |  |  |  |  |  |  |  |  |  |
| **Dose-day data available, N*** | | | 57 | 45 | | 21 | 15 | | | | 6 | 2 | | | 1 | |  | | |  | | |  | | | | |  |  |  |  |  |  |  |  |  |  |  |  |  |  |  |  |  |  |  |  |  |  |  |
| **Missed dose-day, (n/N)%** | | | 11 | 0 | | 0 | 0 | | | | 0 | 0 | | | 0 | |  | | |  | | |  | | | | |  |  |  |  |  |  |  |  |  |  |  |  |  |  |  |  |  |  |  |  |  |  |  |
|  | | |  |  | |  |  | | | |  |  | | |  | |  | | |  | | |  | | | | |  |  |  |  |  |  |  |  |  |  |  |  |  |  |  |  |  |  |  |  |  |  |  |
| **Missed dose-days of oral azithromycin among 26 hospitalized patients who received in-hospital azithromycin, Uganda, 2014** | | | | | | | | | | | | | | | | | | | | | | | | | | | |  |  |  |  |  |  |  |  |  |  |  |  |  |  |  |  |  |  |  |  |  |  |  |
| **Length of stay, days** | **Days missed** | | **Day 1** | **Day 2** | | **Day 3** | **Day 4** | | | | **Day 5** | **Day 6** | | | **Day 7** | | **Day 8** | | | **Day 9** | | |  |  |  |  |  |  |  |  |  |  |  |  |  |  |  |  |  |  |  |  |  |  |  |  |  |  |  |  |
| 3 | 0 | |  |  | |  |  | | | |  |  | | |  | |  | | |  | | |  |  |  |  |  |  |  |  |  |  |  |  |  |  |  |  |  |  |  |  |  |  |  |  |  |  |  |  |
| 3 | 0 | |  |  | |  |  | | | |  |  | | |  | |  | | |  | | |  |  |  |  |  |  |  |  |  |  |  |  |  |  |  |  |  |  |  |  |  |  |  |  |  |  |  |  |
| 3 | 0 | |  |  | |  |  | | | |  |  | | |  | |  | | |  | | |  |  |  |  |  |  |  |  |  |  |  |  |  |  |  |  |  |  |  |  |  |  |  |  |  |  |  |  |
| 4 | 0 | |  |  | |  |  | | | |  |  | | |  | |  | | |  | | |  |  |  |  |  |  |  |  |  |  |  |  |  |  |  |  |  |  |  |  |  |  |  |  |  |  |  |  |
| 4 | 0 | |  |  | |  |  | | | |  |  | | |  | |  | | |  | | |  |  |  |  |  |  |  |  |  |  |  |  |  |  |  |  |  |  |  |  |  |  |  |  |  |  |  |  |
| 4 | 0 | |  |  | |  |  | | | |  |  | | |  | |  | | |  | | |  |  |  |  |  |  |  |  |  |  |  |  |  |  |  |  |  |  |  |  |  |  |  |  |  |  |  |  |
| 4 | 0 | |  |  | |  |  | | | |  |  | | |  | |  | | |  | | |  |  |  |  |  |  |  |  |  |  |  |  |  |  |  |  |  |  |  |  |  |  |  |  |  |  |  |  |
| 4 | 0 | |  |  | |  |  | | | |  |  | | |  | |  | | |  | | |  |  |  |  |  |  |  |  |  |  |  |  |  |  |  |  |  |  |  |  |  |  |  |  |  |  |  |  |
| 5 | 3 | |  |  | |  |  | | | |  |  | | |  | |  | | |  | | |  |  |  |  |  |  |  |  |  |  |  |  |  |  |  |  |  |  |  |  |  |  |  |  |  |  |  |  |
| 5 | 2 | |  |  | |  |  | | | |  |  | | |  | |  | | |  | | |  |  |  |  |  |  |  |  |  |  |  |  |  |  |  |  |  |  |  |  |  |  |  |  |  |  |  |  |
| 5 | 1 | |  |  | |  |  | | | |  |  | | |  | |  | | |  | | |  |  |  |  |  |  |  |  |  |  |  |  |  |  |  |  |  |  |  |  |  |  |  |  |  |  |  |  |
| 5 | 1 | |  |  | |  |  | | | |  |  | | |  | |  | | |  | | |  |  |  |  |  |  |  |  |  |  |  |  |  |  |  |  |  |  |  |  |  |  |  |  |  |  |  |  |
| 5 | 0 | |  |  | |  |  | | | |  |  | | |  | |  | | |  | | |  |  |  |  |  |  |  |  |  |  |  |  |  |  |  |  |  |  |  |  |  |  |  |  |  |  |  |  |
| 5 | 0 | |  |  | |  |  | | | |  |  | | |  | |  | | |  | | |  |  |  |  |  |  |  |  |  |  |  |  |  |  |  |  |  |  |  |  |  |  |  |  |  |  |  |  |
| 5 | 0 | |  |  | |  |  | | | |  |  | | |  | |  | | |  | | |  |  |  |  |  |  |  |  |  |  |  |  |  |  |  |  |  |  |  |  |  |  |  |  |  |  |  |  |
| 6 | 3 | |  |  | |  |  | | | |  |  | | |  | |  | | |  | | |  |  |  |  |  |  |  |  |  |  |  |  |  |  |  |  |  |  |  |  |  |  |  |  |  |  |  |  |
| 6 | 0 | |  |  | |  |  | | | |  |  | | |  | |  | | |  | | |  |  |  |  |  |  |  |  |  |  |  |  |  |  |  |  |  |  |  |  |  |  |  |  |  |  |  |  |
| 6 | 0 | |  |  | |  |  | | | |  |  | | |  | |  | | |  | | |  |  |  |  |  |  |  |  |  |  |  |  |  |  |  |  |  |  |  |  |  |  |  |  |  |  |  |  |
| 7 | 2 | |  |  | |  |  | | | |  |  | | |  | |  | | |  | | |  |  |  |  |  |  |  |  |  |  |  |  |  |  |  |  |  |  |  |  |  |  |  |  |  |  |  |  |
| 7 | 0 | |  |  | |  |  | | | |  |  | | |  | |  | | |  | | |  |  |  |  |  |  |  |  |  |  |  |  |  |  |  |  |  |  |  |  |  |  |  |  |  |  |  |  |
| 9 | 0 | |  |  | |  |  | | | |  |  | | |  | |  | | |  | | |  |  |  |  |  |  |  |  |  |  |  |  |  |  |  |  |  |  |  |  |  |  |  |  |  |  |  |  |
| 9 | 0 | |  |  | |  |  | | | |  |  | | |  | |  | | |  | | |  |  |  |  |  |  |  |  |  |  |  |  |  |  |  |  |  |  |  |  |  |  |  |  |  |  |  |  |
| 9 | 0 | |  |  | |  |  | | | |  |  | | |  | |  | | |  | | |  |  |  |  |  |  |  |  |  |  |  |  |  |  |  |  |  |  |  |  |  |  |  |  |  |  |  |  |
| 10 | 1 | |  |  | |  |  | | | |  |  | | |  | |  | | |  | | |  |  |  |  |  |  |  |  |  |  |  |  |  |  |  |  |  |  |  |  |  |  |  |  |  |  |  |  |
| 11 | 1 | |  |  | |  |  | | | |  |  | | |  | |  | | |  | | |  |  |  |  |  |  |  |  |  |  |  |  |  |  |  |  |  |  |  |  |  |  |  |  |  |  |  |  |
| 11 | 0 | |  |  | |  |  | | | |  |  | | |  | |  | | |  | | |  |  |  |  |  |  |  |  |  |  |  |  |  |  |  |  |  |  |  |  |  |  |  |  |  |  |  |  |
| **Missed dose-day, n** | | | 8 | 4 | | 2 | 0 | | | | 0 | 0 | | | 0 | | 0 | | |  | | |  |  |  |  |  |  |  |  |  |  |  |  |  |  |  |  |  |  |  |  |  |  |  |  |  |  |  |  |
| **Dose-day data unavailable, n1** | | | 0 | 5 | | 8 | 17 | | | | 21 | 23 | | | 25 | | 25 | | |  | | |  |  |  |  |  |  |  |  |  |  |  |  |  |  |  |  |  |  |  |  |  |  |  |  |  |  |  |  |
| **Dose-day data available, N*** | | | 26 | 21 | | 18 | 9 | | | | 5 | 3 | | | 1 | | 1 | | |  | | |  |  |  |  |  |  |  |  |  |  |  |  |  |  |  |  |  |  |  |  |  |  |  |  |  |  |  |  |
| **Missed dose-day, (n/N)%** | | | **31** | **19** | | **11** | **0** | | | | **0** | **0** | | | **0** | | **0** | | |  | | |  |  |  |  |  |  |  |  |  |  |  |  |  |  |  |  |  |  |  |  |  |  |  |  |  |  |  |  |
|  | |  |  |  | |  |  | | | |  |  | | |  | |  | |  |  | | |  | | |  |  | | |  | | | |  |  |  |  |  |  |  |  |  |  |  |  |  |  |  |  |  |

**Table S4. Detailed account of oral azithromycin administration among 26 hospitalized patients who received azithromycin during hospital stay, Uganda, 2014**

| **fileno** | **Missed at**  **least one dose-day** | **Doses**  **completed while in hospital** | **Duration of**  **Administrati on, days** | **Notes** | **Working diagnoses** | **Medication**  **Error Occurred** | **Committed by** | **Outcome** | **Source of the Azithromycin** |
| --- | --- | --- | --- | --- | --- | --- | --- | --- | --- |
| 10 | No | Yes | 6 | 500mg of azithromycin taken twice daily for 6 days  instead of 500mg once-daily for 6 days as prescribed. Patient manifested with azithromycin gastrointestinal tract signs & symptoms | Type 2 Diabetes Mellitus,  Pulmonary Tuberculosis, Lobar  Pneumonia | Yes | Patient | Nausea of moderate severity  suspectedly attributed to excessive dose of Azithromycin | Private community pharmacy |
| 38 | Yes | Yes | 5 | Treatment was prescribed once-daily for 3-days.  Patient missed first dose-day of azithromycin treatment and took five once-daily doses thereafter | Broncho-pneumonia, COPD,  Empyema | Yes | Patient |  | Ward Pharmacy |
| 44 | No | Yes | 3 | Treatment was prescribed once-daily for 5-days.  Patient took only three doses and was discharged. | Malaria in pregnancy, vaginal  candidiadis, UTI | Not known |  |  | Ward Pharmacy |
| 47 | Yes | No | 1 | Treatment was prescribed once-daily for 5-days.  Patient started treatment a day later and took only one once-daily azithromycin dose. Details regarding subsequent doses are not known. | Lobar pneumonia, ISS,  Bronchopneumonia | Yes | Could not be determined |  | Ward Pharmacy |
| 50 | No | Yes | 5 | Treatment was prescribed once-daily for 5-days.  Patient took five doses and was discharged | Lobar pneumonia, Pulmonary  tuberculosis | No |  |  | Ward Pharmacy |
| 71 | No | No | 1 | Treatment was prescribed once-daily for 5-days.  Patient took only one dose, stopped. | Broncho-asthma,  Bronchopneumonia, Pulmonary tuberculosis | Yes | Patient |  | Ward Pharmacy |
| 89 | No | No | 3 | Treatment was prescribed once-daily for 5-days.  Patient took three once-daily doses and was discharged. | Community-acquired  Pneumonia, Bronchopneumonia | Not known |  |  | Private community pharmacy |
| 94 | No | No | 3 | Treatment was prescribed for 5-days (5 doses).  Patient took three doses and was discharged. | HHD, ISS, UTI, Pneumonia | Not known |  |  | Ward Pharmacy |
| 102 | No | No | 2 | Treatment was prescribed for 7-days. Patient took  two doses and was discharged. | Lobar Pneumonia, COPD  exacerbation, Bronchiectasis | Not known |  |  | Ward Pharmacy |
| 111 | No | No | 1 | Treatment was prescribed for 5-days. Patient took  only one dose and was discharged 2-days later | Asthma | Yes | Could not be determined |  | Ward Pharmacy |
| 115 | Yes | Yes | 8 | Treatment was prescribed for 3-days. Patient  swallowed five additional once-daily doses. Ward pharmacist dispensed more doses than were prescribed. One dose (day) was missed after completing four doses and the patient resumed treatment a day afterwards to complete eight doses of azithromycin. | Emphysema, Asthma | Yes | Healthcare  Professional |  | Ward Pharmacy |
| 120 | Yes | No | 1 | Treatment was prescribed for 5-days. Patient  started treatment 2 days later, took a single once- daily dose of azithromycin and stopped - was lost to follow-up three days later. | Pulmonary tuberculosis Relapse,  ISS | Yes | Patient |  | Ward Pharmacy |
| 158 | Yes | No | 3 | Treatment was prescribed 500mg once-daily for 6-  days. Patient started treatment 3-days later and took two doses of 250mg 12 hours apart, then took a further two doses of 250mg 24 hourly before discharge from hospital | ISS, Gastroenteritis, Oral  candidiasis, Bronchopneumonia | Yes | Patient |  | Ward Pharmacy |
| 232 | No | No | 3 | Treatment was prescribed for 5-days. Patient took  only three doses and was discharged | Septicaemia, Typhoid, Anaemia,  Pneumonia | Not known |  |  | Ward Pharmacy |

| **fileno** | **Missed at**  **least one dose-day** | **Doses**  **completed while in hospital** | **Duration of**  **Administrati on, days** | **Notes** | **Working diagnoses** | **Medication**  **Error Occurred** | **Committed by** | **Outcome** | **Source of the Azithromycin** |
| --- | --- | --- | --- | --- | --- | --- | --- | --- | --- |
| 377 | No | No | 2 | Treatment was prescribed for 5-days. Patient took  two doses and was discharged. | ISS, Pulmonary tuberculosis,  Pneumonia | Not known |  |  | Ward Pharmacy |
| 452 | Yes | No | 3 | Treatment was prescribed for 5-days. Patient  started treatment one day later and took three once- daily doses prior to discharge from hospital | Pulmonary tuberculosis, ISS,  Lung Malignancy, Massive  Pleural Effusion | Yes | Could not be determined |  | Ward Pharmacy |
| 510 | No | No | 2 | Treatment was prescribed for 5-days. Patient took  two doses and was discharged. | ISS, Bronchopneumonia | Not known |  |  | Private community pharmacy |
| 574 | Yes | No | 2 | Treatment was prescribed for 5-days. Patient  started treatment a day later and took two once daily doses before running away from hospital. | Pulmonary tuberculosis,  Septicaemia, Malaria, Pleural  Effusion | Yes | Could not be determined |  | Private community pharmacy |
| 575 | No | No | 4 | Treatment was prescribed for 7-days. Patient took  four doses and was discharged. | Cardiac Asthma, Ischaemic  cardiomyopathy | Not known |  |  | Private community pharmacy |
| 598 | No | No | 1 | Treatment was prescribed for 5-days. Patient took  one dose and was discharged. | ISS, Malaria, Septicaemia,  Chronic Bronchitis | Not known |  |  | Ward Pharmacy |
| 609 | No | -- | 3 | Duration of treatment was not indicated on  prescription by the medical doctor (prescriber). | Constrictive pericarditis, Left  and Right Heart Failure, Pleural  Effusion | Yes | Healthcare  Professional |  | Private community pharmacy |
| 623 | Yes | No | 2 | Treatment was prescribed for 5-days. Patient  started treatment 2-days later and took two once- daily doses before being discharged. | Pulmonary tuberculosis, HIV-  induced Psychosis, CCM, Bacterial Pneumonia, PCP, Malaria | Yes | Could not be determined |  | Not Known |
| 624 | No | No | 1 | Treatment was prescribed for 5-days. Patient took  one dose and was switched to erythromycin | COPD, Sepsis, Bronchitis,  Pneumonitis, Hepatitis, Pulmonary tuberculosis, Bronchial asthma, Col Pulmunale | No |  |  | Private community pharmacy |
| 690 | No | No | 1 | Treatment was prescribed for 5-days. Patient took  one dose and was discharged. | Lobar Pneumonia, Rule-out  Pulmonary tuberculosis, LRTI, Right Pleural Effusion | Not known |  |  | Ward Pharmacy |
| 729 | No | No | 3 | Treatment was prescribed for 5-days. Patient took  only three doses once-daily and was discharged. | Chronic Bronchitis, Pulmonary  tuberculois, Asthma | Not known |  |  | Ward Pharmacy |
| 731 | No | No | 1 | Treatment was prescribed for 5-days. Patient took  only one dose and stopped - was discharged three days later | Bronchopneumonia, Pulmonary  tuberculosis, Multi-drug  Resistant tuberculosis | Yes | Patient |  | Ward Pharmacy |

| **Table S5. Excess of administered over dispensed intravenous ceftriaxone doses** | |
| --- | --- |
| **Reason No.** | **Explanation** |
| 1 | The hospital sometimes run out of ceftriaxone stocks and patients sourced the drug from private retail pharmacies. Mulago Hospital does not provide for documentation of dispensing information for drugs bought from private community pharmacies, and this dispensing information was not reliably disclosed to research teams when patients/caregivers or ward staff were interviewed. Ward nurses frequently administered, to patients, the purchased intravenous ceftriaxone prior to/after visits by the research teams, who made a minimum of two daily visits to each patient (one early morning visit before the daily ward round – mainly to capture clinical complaints experienced during the previous night since the last visit by the research team plus any new drug information, and another visit after the ward around – to capture new clinical and drug information decisions made by the ward staff). It was not always possible for patients/caregivers to recall fully (oral) drugs taken during the times that research teams were away (at night) and this was especially so if patients were very ill and/or if patients/caregivers did not remember details of administered drugs. However, a 12- to 14-hour overnight recall period was sufficiently short for most patients/caregivers to remember administered medicines. Research teams spent 8-10 hours on the wards each day during which they periodically returned to patients to obtain any previously uncaptured/pending patient information; |
| 2 | That the ward pharmacists did not record dispensed ceftriaxone – but this was likely to be negligible since it was mandatory for pharmacists to provide accountability of dispensed medicines before requisitioning new stock and they thus have an incentive to maintain up-to-date medicines dispensing records; |
| 3 | Ward nurses administered ceftriaxone treatment using emergency stock in their drug trolleys and did not follow-up with adequate documentation of the dispensed drug. This information was rather difficult to track by the research team; |
| 4 | Undisclosed prescription and administration of intravenous ceftriaxone to patients by HCPs other than the ward staff, e.g. doctor or nurse friend; |
| 5 | Inadvertent failure by the research team to record otherwise available ceftriaxone dispensing information. |

| **Figure S1. Computation of missed-dose days of antibiotic prescribing** | | | | | | | | | | | | |  |
| --- | --- | --- | --- | --- | --- | --- | --- | --- | --- | --- | --- | --- | --- |
|  |  |  |  |  |  |  |  |  |  |  | |  | |
| **Options** |  |  |  |  |  |  |  |  |  |  | | **Notes** | |
| Option 1: |  | **P** | 🞭 | 🞭 | **ǁ** |  |  |  |  |  | | The time-lag between day of prescription and first dose-day was 1-day. Thus, patient missed one dose-day since drug was started the following day. | |
|  |  |  |  |  |  |  |  |  |  |  | |  | |
|  |  |  |  |  |  |  |  |  |  |  | |  | |
| Option 2: |  | **P** | 🞭 | 🞭 | 🞍 | 🞭 | **ǁ** |  |  |  | | Patient missed one dose-day on the day of prescription since drug was started the following day.  Patient also missed Day three of treatment. Total: 2 missed dose-days. | |
|  |  |  |  |  |  |  |  |  |  |  | |  | |
|  |  |  |  |  |  |  |  |  |  |  | |  | |
| Option 3: |  | **P** | 🞭 | 🞭 | 🞍 | 🞭 | 🞭 | 🞭 | **ǁ** |  | | Missed dose-days were 2 out of 7 days over which administration of medication was stretched despite having been prescribed treatment for 5-days. | |
|  |  |  |  |  |  |  |  |  |  |  | |  | |
|  |  |  |  |  |  |  |  |  |  |  | |  | |
| Option 4: |  | **p, X** | 🞭 | 🞍 | 🞭 | 🞭 | 🞭 | **ǁ** |  |  | | The time-lag between day of prescription and first dose-day was zero. Missed dose-days were 1 out of 6 days over which treatment was stretched despite 5-day prescription. | |
|  |  |  |  |  |  |  |  |  |  |  | |  | |
|  |  | **Assumptions for this illustration:** | | | | | |  |  |  | |  | |
|  |  | 1) Antibiotic was prescribed for 5-days | | | | | | |  |  | |  | |
|  |  | 2) We would expect the patient to start treatment on the same day of prescription. | | | | | | | |  | |  | |
|  |  | 3) Patient did not receive antibiotic on the day of discharge although this was not the case for most patients | | | | | | | | | | |  |
|  |  |  |  |  |  |  |  |  |  |  |  | |  |
|  |  | **KEY** |  |  |  |  |  |  |  |  |  | |  |
|  |  | **P** | Day of prescription of antibiotic | | |  |  |  |  |  |  | |  |
|  |  | 🞭 | Day that an antibiotic was administered = one dose-day | | | | |  |  |  |  | |  |
|  |  | **ǁ** | Discharged from ward | | | | |  |  |  |  | |  |

**Supplementary Results: Prescription, dispensing and administration of frequently used hospital-initiated antibiotics**

*Ceftriaxone*: Only 62% (1178/1895; 95% CI: 60% to 64%) of prescribed ceftriaxone doses were administered and only 66% (783/1178; 95% CI: 64% to 69%) of administered ceftriaxone doses were accounted for from available information on dispensed ceftriaxone doses. Seventy-four percent (273/371; 95% CI: 63% to 85%) of inpatients received fewer ceftriaxone doses than were prescribed, see **Table 4** & **5**.

*Metronidazole*: Only 27% (1043/3862; 95% CI: 26% to 28%) of prescribed metronidazole doses and 64% (1043/1642; 95% CI: 62% to 66%) of dispensed metronidazole doses were administered. Also, 97% (222/230; 95% CI: 93% to 98%) of patients received fewer metronidazole doses than were prescribed, see **Table 4** & **5**.

*Ciprofloxacin*: Only 35% (396/1130; 95% CI: 32% to 38%) of prescribed ciprofloxacin doses and 54% (396/728; 95% CI: 50% to 58%) of dispensed ciprofloxacin doses were administered. Out of 105 (of 114) patients in whom full information on in-hospital prescribed ciprofloxacin was available, 90% (94/105; 95% CI: 84% to 96%) received fewer doses than were prescribed, see **Table 4** & **5**.

*Azithromycin*: Medication errors were observed in half (13/26; 95% CI: 31% to 69%) the patients who received oral azithromycin and seven of the 26 (27%) patients purchased azithromycin from a private community pharmacy. One patient took double the recommended daily dose of azithromycin for six days which might have resulted in a suspected ADR: non-serious moderately severe nausea. Six medication administration errors were committed by patients while one medication dispensing error was made by a ward pharmacist and one prescription error by a medical doctor, see **Table S3 & S4**.

**Supplementary Discussion: Drug administration issues**

Clinical ward rounds manned by a team of doctors (specialist/consultant, senior house officer, and two interns), an intern pharmacist, and two nurses typically take five to six hours, usually commencing at 9am till 2-3pm. Duration is dependent on the capacity of the ward-team to review the 50-80 inpatients, sometimes in the absence of the consultant. At the end of each round, the nurses and intern pharmacist turn to the patients’ files to review the prescribed medications. Ideally, pharmacist interns dispense parenteral medications at the bedside for nurse-administration, directly. Frequently, however, neither the patients nor the nurse (from emergency stock) readily have the prescribed parenteral medications for immediate administration. On such occasions, the on-duty nurse advises the patient/caregiver to pick-up the prescribed parenteral medicines from the ward pharmacy using the prescription form in the patient’s file. Prescribed oral medications are primarily dispensed at the ward pharmacy where the patient/caregiver obtains the medicines although this arrangement makes it difficult for the very sick patients to receive their medications in the absence of their caregiver. In addition, the day working shift (8am to 3pm) oftentimes ends before ward staff have dispensed/administered the prescribed medicines to all ward-round reviewed patients. Thus, nurses and intern pharmacists in the subsequent working shifts (3pm to 9pm; or 9pm to 8am) handle the dispensing/administration of medicines, which creates further delays in the initiation of treatment and/or increases the risk to the inpatients of missing/failing to continue with prescribed treatment.
